# *In situ* electro-organic synthesis of hydroquinone using anisole on MWCNT/Nafion modified electrode surface and its heterogeneous electrocatalytic reduction of toxic Cr(vi) species†

**DOI:** 10.1039/d0ra10370e

**Published:** 2021-01-20

**Authors:** Mansi Gandhi, Desikan Rajagopal, Annamalai Senthil Kumar

**Affiliations:** Nano and Bioelectrochemistry Research Laboratory, Department of Chemistry, School of Advanced Sciences, Vellore Institute of Technology University Vellore-632014 India askumarchem@yahoo.com askumar@vit.ac.in +91-416-220-2754; Department of Chemistry, School of Advanced Sciences, Vellore Institute of Technology University Vellore-632014 India rajagopal.desikan@vit.ac.in +91-407-590-3978 +91-416-220-2330; Carbon Dioxide Research and Green Technology Centre, Vellore Institute of Technology University Vellore-632014 Tamil Nadu India

## Abstract

Owing to its electro-inactive character, anisole (phenylmethyl ether, PhOCH_3_) and its related derivatives have been used as electrolytes in electrochemistry. Herein, we report a simple one-step electro-organic conversion of PhOCH_3_ to hydroquinone (HQ) on a pristine-MWCNT–Nafion modified electrode glassy carbon electrode surface, GCE/Nf–MWCNT@HQ, in pH 2 KCl–HCl solution within 15 min of working time. The chemically modified electrode showed a highly redox-active and well-defined signal at an apparent standard electrode potential, *E*^o^′ = 0.45 V *vs.* Ag/AgCl (A2/C2) with a surface excess value, *Γ*_HQ_ = 2.1 × 10^−9^ mol cm^−2^. The formation of surface-confined HQ is confirmed by collective physicochemical and spectroscopic characterizations using TEM, UV-Vis, Raman, FTIR, NMR and GC-MS techniques and with several control experiments. Consent about the mechanism, the 2.1% of intrinsic iron present in the pristine-MWCNT is involved for specific complexation with oxygen donor organic molecule (PhOCH_3_) and hydroxylation in presence of H_2_O_2_ (nucleophilic attack) for HQ-product formation. The GCE/Nf–MWCNT@HQ showed an excellent heterogeneous-electrocatalytic reduction of Cr(vi) species in acidic solution with a linear calibration plot in a range, 5–500 ppm at an applied potential, 0.4 V *vs.* Ag/AgCl with a detection limit, 230 ppb (S/N = 3; amperometric *i*–*t*). As a proof of concept, selective detection of toxic Cr(vi) content in the tannery-waste water has been demonstrated with a recovery value ∼100%.

## Abstract 2

MWCNT-surface confined Hydroquinone/quinone redox system is prepared *in situ* method and used for mediated reduction of Cr(vi) species

## Graphical abstract

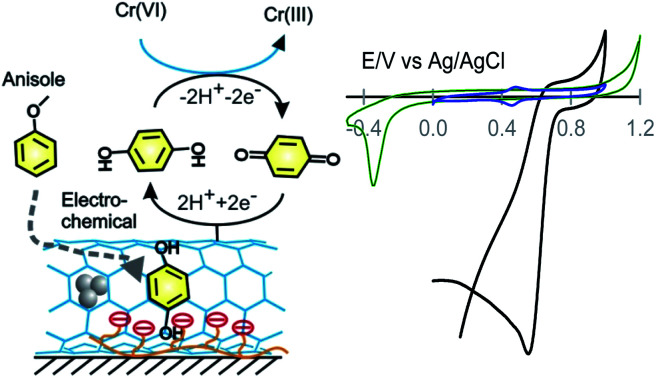

## 1. Introduction

Enzyme assisted hydroxylation of aromatic compounds to their respective phenolic and polyphenolic derivatives has been referred to as an important synthetic reaction in organic chemistry.^1,2^ Hydroxylation of aromatic organic compounds by cytochrome c, horseradish peroxide, tyrosinase enzymes, and certain bacterial based systems in the presence of a dilute concentration of H_2_O_2_ have been used as a conventional synthetic reaction.^1^ The role of the intermediate peroxy radical species and the iron-porphyrin active site of the enzyme-catalysts are key factors in the reaction.^1,2^ Bio-mimicking the functionality and understanding its mechanism is a long-standing research interest. In 1954, Udenfriend *et al.* first reported a biomimicking hydroxylation of aromatic compounds based on a reaction mixture composed of iron(ii), ascorbic acid (AA), ethylene diamine tetraacetic acid (EDTA) and H_2_O_2_ in a neutral pH solution.^3^ It was claimed that the {HOO–Fe(ii)–AA–EDTA} complex formed as an intermediate species was involved in the transformation of an oxygen atom from the HOO–Fe species to the aromatic compound.^3^ In 1963, Hamilton and Friedman replaced the AA in the reaction mixture with hydroquinone (HQ) or catechol (CA) and demonstrated the hydroxylation of methoxy benzene (anisole) by H_2_O_2_. It has been revealed that hydroxylating species is an electrophilic system (˙OOH is linked with the reactant species in the intermediate step),^4^ without the association of any free hydroxyl radical species (Fenton reagent). Such a procedure has shown about four times higher product conversion efficiency than that of the regular Fenton reaction. Similar to the above observation, several metal oxide/complexes along with H_2_O_2_ have been reported for the hydroxylation of aromatic compounds. Following are some of the representative examples for the catalytic system coupled with H_2_O_2_: methylrhenium trioxide,^5^ Ru/Ir,^6^ and without H_2_O_2_ are Ru based cyclononane,^7^ Ru and Fe on titania supported,^8^ CNT–Pd/TiO_2_, CNT–Cu/TiO_2_,^9^ FeReO_*x*_/ZrO_2_,^10^ Mn(iv) and Fe(ii)^11^ oxides, Ni/SiO_2_,^12^ zeolite (HBeta, HY),^13^ MoO_3_/ZrO_2_,^14^ zeolite ZSM-5,^15^ zeolite HZ-25,^16^ P–Fe/SiO_2_,^17^ Hg(ii),^18^*etc.* First time in this work, we report, an *in situ* electrochemical reaction assisted demethoxylation and hydroxylation of an aromatic ether, anisole (PhOCH_3_) to hydroquinone (HQ) on intrinsic iron impurity containing multiwalled carbon nanotube/Nafion (Nf–MWCNT) chemically modified electrode surface in mild acidic condition.

Owing to the non-amenable character, PhOCH_3_ and its related derivatives have been used as a universal green-solvent similar to ethanol and water for various chemical^19–21^ and electrochemical reactions.^22^ There are few reports for the electrochemical amination of PhOCH_3_ in a strong acidic condition with hydroxylamine as an aminating reagent.^23–25^ In this report, an Nf–MWCNT modified glassy carbon electrode, designated as GCE/Nf–MWCNT, has been used as a specific electrode system for electrochemical molecular conversion of PhOCH_3_ to highly-redox active surface-confined HQ, *i.e.*, HQ immobilized on Nf–MWCNT, *i.e.*, GCE/Nf–MWCNT@HQ, in presence of a dilute H_2_O_2_ solution. Intrinsic iron-containing pristine-MWCNT is found to be the best carbon nanomaterial compared to the other carbon nanomaterials in this work. Nafion used in this work helped to turn the buried iron oxide as iron ion species and further complexation with the organic compound and H_2_O_2_ species.

In general, Fe, Ni and Co-based metallic systems have been used as a catalyst for the preparation of carbon nanotubes.^26–29^ Due to the inherent physical property, the fraction (∼5–20%) of the above catalyst species are carried over into the final product.^26^ A specific role of the iron impurity in MWCNT, in particular, iron-mediated electrochemical oxidation and reduction reactions of certain electroactive-compounds like H_2_O_2_, cysteine (CySH), hydrazine have been reported in the literature.^26–28,30^ The 2–4 wt% iron oxide impurity aided conjugation of new motifs on the multiwall as well.^26,30^ In this regard, our group has reported *in situ* derivatization of the intrinsic iron impurity in the MWCNT as a surface-confined redox system, MWCNT–Fe(bpy)_3_^2+^, and in another case, as a transducer for molecular wiring with hemoglobin in neutral pH solution.^26^ In addition, pristine-MWCNT as an intrinsic electrochemical-Fenton reagent for conversion of benzene to a surface-confined mixture catechol (1,2-dihydroxybenzene) and HQ in presence of 260 mM of H_2_O_2_ pH 2 KCl–HCl solution.^27^ Interestingly, in this work, we first time identified a unique electro-organic conversion of PhOCH_3_ to HQ on Nf–MWCNT surface in presence of dilute H_2_O_2_. The modified electrode, Nf–MWCNT@HQ prepared within 15 min time, has shown a selective electrocatalytic reduction function of toxic hexavalent chromium (Cr(vi)) species *via* mediated reduction mechanism in pH 2 HCl–KCl buffer.

Chromium ion is a potentially toxic heavy metal system present in soil and water bodies as an environmental pollutant.^31,32^ It exists as trivalent (Cr(iii)) and hexavalent (Cr(vi)) and/or Cr(vi) species in the environment. The toxicity of chromium compounds is primarily due to Cr(vi) species, whereas, Cr(iii) is a non-toxic substance and a nutrient to the physiological system, for instance, insulin action.^32^ The Cr(vi) species can be easily absorbed by the large intestine and gastrointestinal tract and enter into the cellular system. Under physiological conditions, the Cr(vi) species can oxidize several biochemicals like H_2_O_2_, glutathione, ascorbic acid, DNA, proteins, and membrane lipids, thereby disrupting cellular functions.^33^ Occupational exposure to the toxic chromium(vi) species in industries has been associated with an increased risk of respiratory system cancers. Health agencies like the World Health Organization (WHO) and the United States Environmental Protection Agency *etc.*,^34^ have barred the maximum permissible level for Cr species into the environment to be <50 ppm. Numerous analytical methods of Cr speciation and quantification involve the use of spectrophotometry,^35^ colorimetric,^36^ hyper Rayleigh scattering,^37^ luminescence,^38^ cation–anion based column chromatography,^39,40^ high-performance liquid chromatography inductively coupled mass spectroscopy^41–44^*etc.*, have been reported. Electrochemical techniques are considered to be a simple, direct, and skilled technician-free approach for practical applications.^45–47^ In this connection, Pt and Au nanoparticle-based chemically modified electrodes have often been worked as a sensor for this purpose.^48,49^ Indeed, due to the high cost of the precious metals and surface-fouling of the chemically modified electrodes restrict the electrochemical procedures for further electroanalytical applications. Alternately, mediators modified electrodes like MWCNT/quercetin–SPCE,^46^ Chit/MWCNT@MnO_*x*_-nanoparticle,^50^ AuNP/polyaniline-*co*-polytoluidine (PoT),^51^ g-C_3_N_4_/Ag/Nf/GCE,^52^ polyoxometalate^53^ and ZnO–Fe_2_O_3_ (ref. 54) *etc.* (Table 1), have been reported as efficient systems. Thus, the development of a simple, stable and surface-fouling-free chemically modified electrode is a challenging task for the Cr(vi) reduction and sensing reaction. A GCE/Nf–MWCNT@HQ modified glassy carbon electrode developed by a simple one-step approach has shown an excellent electrocatalytic reduction of Cr(vi) in pH 2 KCl–HCl solution without any above-mentioned problems. Note that, as a homogeneous catalyst, hexavalent chromium species has been reported for the alcohol derivatives oxidation reaction to respective carbonyl compounds.^55,56^ In this work, the redox-active polyphenol, HQ modified electrode has been used as a heterogeneous-electrocatalyst for the Cr(vi) reduction reaction in an acidic solution. Industrial wastewater effluent-real samples were quantitatively determined using this new electrode by standard addition approach. This work covers, preparation, details characterizations and electrocatalytic Cr(vi) application of redox-active HQ-chemically modified electrode. The prime novelty of this paper is to convert the electro-chemically inactive anisole to electro-active HQ in sitully on MWCNT surface and further use it as electrocatalyst for heterogeneous Cr(vi) reduction and amperometric *i*–*t* sensing reactions. To the best of our knowledge, this is the first electrochemical study for the molecular transformation of anisole to HQ.

**Table 1:** Comparison of various literature based on various CMEs with the present system for the electrochemical reduction and sense of Cr(vi) species^a^

	Chemically modified electrode	pH, electrolyte	Tech.	*E*_pa_/*V vs.* Ag/AgCl	Sensitivity, μA μM^−1^	Linear range (μM)	Ref no.
1	SPCE/MWCNT@QH_2_	pH 6, acetate buffer	ASV	−0.85	0.433	1–106	46
2	GCE/Chit/MWCNT@MnO_*x*_NP	pH 6, acetate buffer	CV	0.60	0.018	0.3–20	50
3	Au/AuNP's/PANI-*co*-PoT/GO	pH 2, HCl	CV; SWV	0.4	—	5–500	52
4	GCE/graphene/AuNPs/composite	pH 1, HClO_4_	CV; amp *i*–*t*	0.20	—	0–20	48
5	GCE/CNF–Chit@AuNano	pH 4, PBS	CV	−0.10	0.041	3–20	47
6	Au/PET	pH 2, 0.1 M HCl	CV; amp *i*–*t*	0.45	0.009	0.1–2	69
7	GCE/polyoxometalate	pH 0.3, H_2_SO_4_	CV; amp *i*–*t*	−0.05	0.117	2–261	53
8	GCE/ZnO–Fe_2_O_3_	pH 7, PBS	CV; DPV	0.20	—	3–30	54
9	GCE/MWCNT–Nf@HQ	pH 7, PBS	CV; amp *i*–*t*	0.40	0.039	15–160	Our work

a QH_2_ = quercetin; ASV = adsorptive stripping voltammetry; Chit = chitosan; PANI-*co*-PoT = poly(aniline-*co*-toluidine); GO = graphene oxide; AgM = silver molybdate; Nf = Nafion; PET = polyethylene terephthalate. Note: GCE/MWCNT–Nf@HQ = GCE/MWCNT–Nf@PhOCH_3_-Redox.

## 2. Experimental section

### 2.1 Materials

Anisole (anhydrous, 99.7%, CAS 100-66-3, Sigma Aldrich), graphitized mesoporous carbon (GMC, 99.95%; <50 nm), graphene oxide (single-layered; SLGO, >80%, 0.6–1.2 nm thickness, 0.5–2.0 μm flake size), single-walled carbon nanotube (SWCNT; ∼70% purity on carbon basis, size 0.7–1.1 nm diameter), COOH functionalized multi-walled carbon nanotube (f-MWCNT, >80% carbon basis; >8% carboxylic group functionalization), double-walled carbon nanotube (DWCNT; 50–80% carbon basis), MWCNT (∼95% purity assay; outer diameter size, 10–15 nm; inner diameter size, 2–6 nm; length, 0.1–10 mm, ∼4% metal (Fe 2.1%; Co ∼1%, Ni ∼1.8%) impurities).^26,29,57^ Nafion (5 wt%; perfluorinated resin solution; Sigma Aldrich) was used in appropriate concentration. 0.1 M pH 2 buffer prepared by appropriate HCl & KCl addition which was further used as a supporting electrolyte throughout this study. Hydrogen peroxide was incurred from Rankem, India and stored in a dark bottle at 5 ± 2 °C. Since dissolved oxygen or nitrogen is not an interfering species, there was no effort taken to remove oxygen or add nitrogen to the reaction mixture. The temperature for the reaction was kept constant the same as room temperature *i.e.* 28 ± 2 °C.

### 2.2 Apparatus

CHI electrochemical workstation model 660C, USA instrument was used to carry the cyclic voltammetric, potentiostatic polarization techniques using 10 mL of working volume using a three-electrode configuration system. A modified form of glassy carbon electrode (GCE; 0.0707 cm^2^ area) was used as a working electrode, Ag/AgCl (kept in 3 M KCl) as a reference electrode and platinum as a counter electrode was used with an appropriate Teflon-cell top and holder setup. The Gas Chromatography-Mass Spectroscopy (GC-MS) for column filtered extracted ethanolic solution was performed using an Agilent 7890B Gas Chromatograph instrument. The proton NMR was recorded on Bunker 400 MHz using CDCl_3_ as a solvent. For the NMR analysis, an extracted solution obtained by sonicating the modified electrode in 500 μL of ethanol and filtered using syringe filter (0.2 μm) was used. A Horiba XploRA instrument, France was used for the Raman spectroscopic analysis of the modified electrodes at a fixed wavelength = 532 nm. UV-Vis was performed with JASCO V-670 Spectrophotometer, Japan using a clear dissolved ethanolic solution. FTIR spectroscopic measurements were performed using Shimadzu (Japan) and UV-Vis spectroscopic studies were done using JASCO (V-670 PC, Japan) 4100 instrument with KBr. Transmission Electron Microscope (TEM) characterization was done by FEI-Technai G2 20 Twin Instrument using a 3 mm diameter copper grid system.

### 2.3 *In situ* preparation of HQ chemically modified electrode

In first, the GCE was polished using an alumina powder kit, rinsed with a copious amount of distilled water and sonicated in ethanol for 1 min to remove suspended impurities, particles and abrasive materials from the polished electrode surface. The electrode is pretreated (−0.2 to 1.2 V *vs.* Ag/AgCl at a scan rate (*v*) of 50 mV s^−1^ for 20 cycles) in pH 2 KCl–HCl before initiating the experiment. 5 μL of 2 mg different carbon allotropes such as CB, GMC, SWCNT, GO, DWCNT, f-MWCNT, MWCNT dispersed in 500 μL of Nafion–ethanol (100 : 400 of 5% Nafion stock solution) was drop-casted on GCE and left for drying in room-temperature (28 ± 2 °C) for 2 ± 1 min. Then, the carbon–Nafion modified GCE was immersed in a cell containing 50 mM Ph-OCH_3_ + 6 mM H_2_O_2_ in pH 2 HCl–KCl solution and potential cycled in a window, 0 to 1 V *vs.* Ag/AgCl for 20 segments at *v* = 50 mV s^−1^. This chemically modified electrode preparation requires 13.3 min only. The electro-activity was observed with major peaks *i.e. E*_pa_ ∼ 0.70 (A1) and *E*^o^′ = 0.450 ± 0.005 V *vs.* Ag/AgCl (A/C2) in the cyclic voltammetric (CV) experiment. The modified electrode was washed with double-distilled H_2_O and medium transferred to a blank pH 2 HCl–KCl solution to obtain a well-defined and stable A2/C2 redox peak.

### 2.4 Procedure for electrocatalytic reduction of dichromate species

**Scheme 1:**
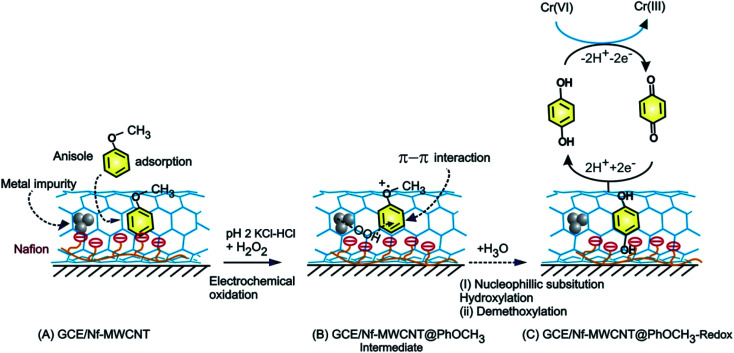
Illustration for the electrochemical π-adsorption of anisole on MWCNT–Nafion modified electrode (A), electrochemical oxidative intermediate (B) and HQ-chemically modified electrodes (C).

The GCE/Nf–MWCNT@HQ modified electrode with the same potential window parameters of CV was used to measure hexavalent chromium species reduction response at *v* = 10 mV s^−1^ as shown in Scheme 1. The amperometric *i*–*t* technique was adopted to study the stepwise decrement with respect to each 5 ppm spike of Cr_2_O_7_^2−^ for 1200 s at an applied potential, *E*_app_ = 0.4 V *vs.* Ag/AgCl for CME with necessary controls and further its interference study were carried out using other interfering analytes. In this work, two scan rates, 10 and 50 mV s^−1^ were used for the voltammetric studies. Since, there is no qualitative changes in the mechanism of the electrochemical reaction, for convenience sake, we fix the scan rate, 50 mV s^−1^ for general studies. Indeed, owing to the maximum current efficiency at a low scan rate, scan rate 10 mV s^−1^ has been chosen as an optimal for the electrocatalytic system.

### 2.5 Real sample analysis

The fresh samples were brought from a local industrial waste discharge (Ranipet, India) which was stored in brown reagent glass bottles. The samples were analyzed with a dilution of 1 : 4 (wastewater : buffer) using the GCE/Nf–MWCNT@HQ modified platform at an *E*_app_ = 0.4 V *vs.* Ag/AgCl using standard addition approach.

## 3. Results and discussion

### 3.1 Electrochemical reaction of PhOCH_3_ on Nafion/pristine MWCNT surface

**Fig. 1:**
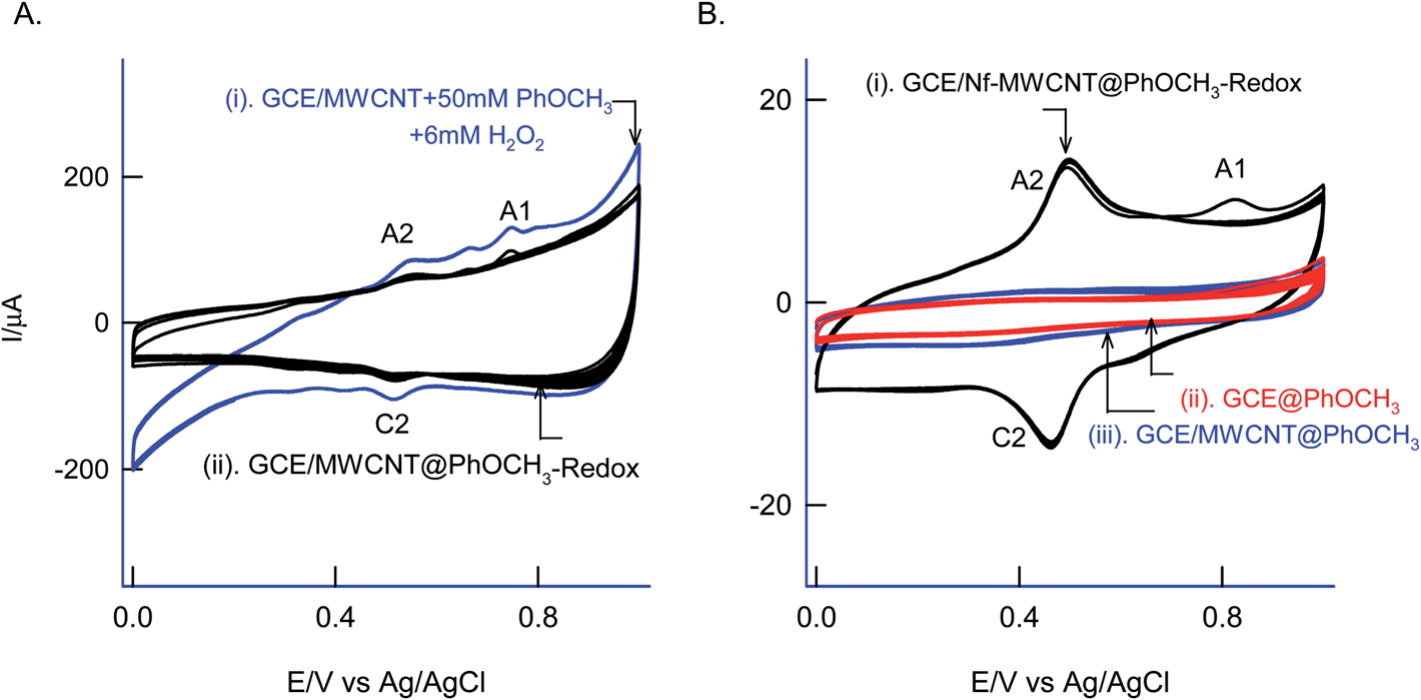
Twenty continuous CV segments of 50 mM PhOCH_3_ dissolved in 10 mL pH 2 KCl–HCl buffer + 6 mM H_2_O_2_ (A) GCE/MWCNT (curve (i)) while its blank transferred as curve (ii). (B) Comparitative CV blank transferred response of GCE (curve (i)) and GCE/MWCNT (curve (ii)) in 50 mM PhOCH_3_ + 10 mL pH 2 KCl–HCl. (B) Curve (iii) is CV of GCE/Nf–MWCNT in 50 mM PhOCH_3_ + 6 mM H_2_O_2_ + 10 mL pH 2 KCl–HCl buffer at *v* = 50 mV s^−1^. Note: Nf = Nafion.

For understanding the electrochemical feature of PhOCH_3_, initial experiments were carried out with a conventional solid electrode, GCE in pH 2 KCl–HCl buffer solution. (ESI Fig. S1A†), is twenty continuous CV responses of an unmodified GCE in 50 mM PhOCH_3_ containing pH 2 KCl–HCl solution. No faradaic response was noticed in the investigated potential window, 0–1.0 V *vs.* Ag/AgCl. To test for any adsorption of the reaction product, after the experiment, the working GCE electrode was transferred to an electrolyte solution and performed the CV (Fig. 1B, curve (ii)). If any electro-active species adsorbed, a specific faradaic signal will be noticed. There is no faradaic response noticed ascribing that the PhOCH_3_ is not an amenable chemical for any electrochemical studies on the conventional electrode surface. This is the reason why the PhOCH_3_ and its relative derivatives have been used as an electrolyte in electrochemical studies.^20–22^ Upon performing the same experiment with GCE/MWCNT as a working electrode, a similar non-faradaic activity was observed, as in (ESI Fig. S1B†) and (Fig. 1B, curve (iii)). Besides, an irreversible back-ground response at 0–0.4 V *vs.* Ag/AgCl was noticed, which may be due to PhOCH_3_ adsorption and stripping on the working electrode surface. Interestingly, when the same experiment was repeated with a mixture of 50 mM PhOCH_3_ + 6 mM H_2_O_2_, a unique irreversible anodic peak at *E*_pa_ = 0.70 V (A1; irreversible) along following with predominant redox peak an apparent standard electrode potential, *E*^o^′ = 0.45 ± 0.005 V *vs.* Ag/AgCl (A2/C), were noticed (Fig. 1A, curve (i)). After being medium transferred to a blank pH 2 KCl–HCl, the modified electrode has retained the electrochemical activity, but with a feeble current output (Fig. 1A, curve (ii)). In further, as a trial and error basis, Nafion–MWCNT composite modified GCE, GCE/Nf–MWCNT has tested the PhOCH_3_ electrochemical reaction as in (ESI Fig. S1C†). This time, well-defined and growth like A1 and A2/C2 redox peaks were noticed. Ten continuous CV of the modified electrodes showed a relative standard deviation (RSD) value 1.8% indicating good stability of the redox-system modified electrode (Fig. 1B, curve (i)). Calculated surface excess, *Γ* = 2.1 × 10^−9^ mol cm^−2^. This value is about 20 times higher than the value of a monolayer configuration, 1 × 10^−10^ mol cm^−2^ indicating a multi-layered configuration system of the modified electrode. Since the precise detail about the redox peak is unknown, the modified electrode is tentatively designated as GCE/Nf–MWCNT@PhOCH_3_-Redox, wherein, PhOCH_3_-Redox = electrochemically oxidized and reduced product of PhOCH_3_.

**Fig. 2:**
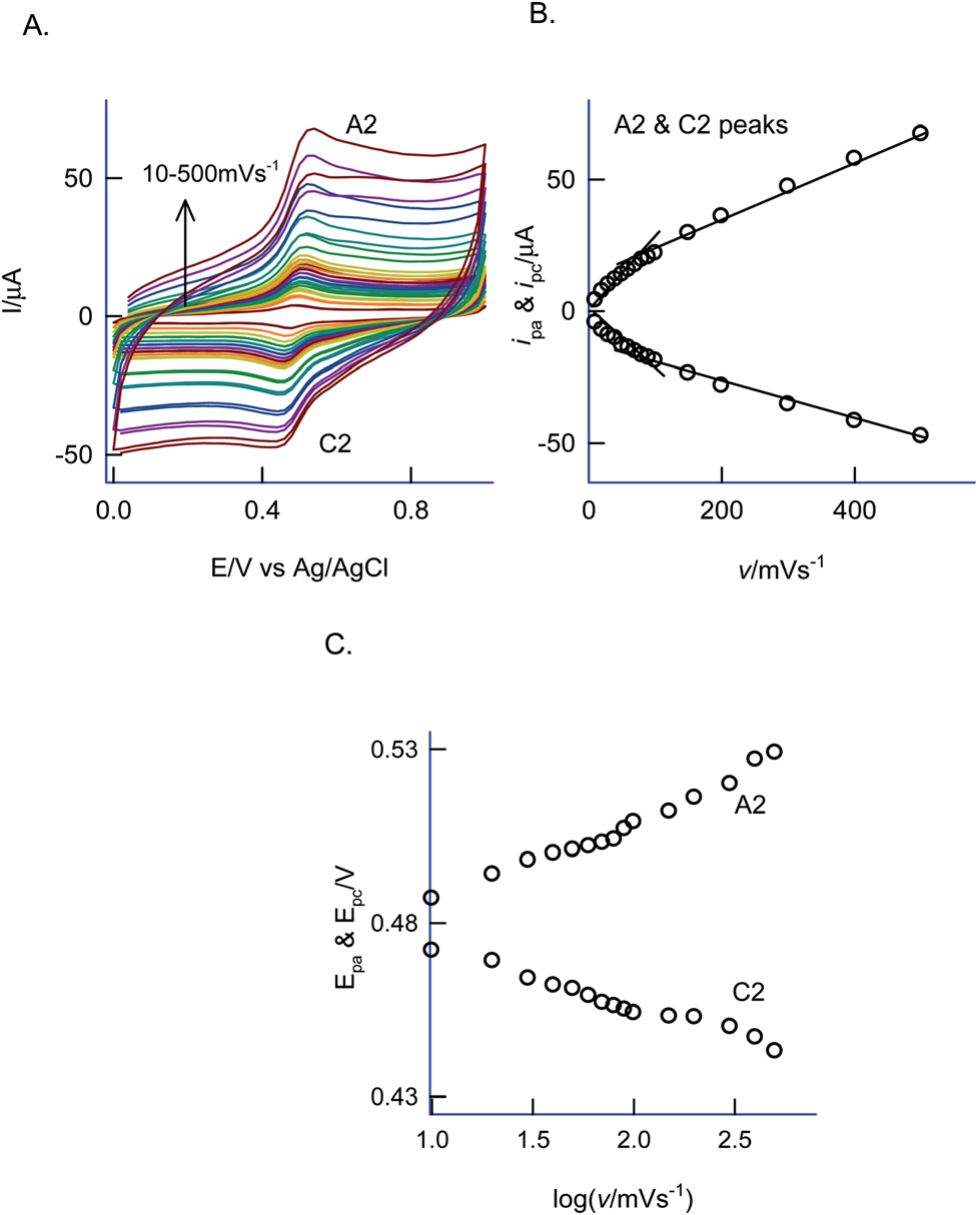
(A) CV response of effect of scan rate (10–500 mV s^−1^) on GCE/Nf–MWCNT@PhOCH_3_-Redox in pH2 KCl–HCl buffer. Plots of (B) anodic peak current (*i*_pa_) and cathodic peak current (*i*_pc_) *vs.* scan rate and *E*_pa_ and *E*_pc_*vs.* log of scan rate (C) for A2/C2 peak.

The electron-transfer feature of the GCE/Nf–MWCNT@PhOCH_3_-Redox was tested by performing an effect of the scan rate in a blank pH 2 KCl–HCl solution. As can be seen in Fig. 2A, a regular increase in the A2/C2 peak current signal against an increase in the scan rate was noticed. Fig. 2B, is a plot of redox peak current signal against the scan rate. Two linear lines were noticed both for anodic and cathodic cases. Considering the nature of the chemically modified electrode, adsorption controlled electron-transfer redox feature with current linearity of *i*_pa_ and *i*_pc_*versus* scan rate starting from the origin is expected. Besides, the obtained non-linearity in the high scan rate region (>50 mV s^−1^) denoting that mixed kinetics, *i.e.*, both adsorption and diffusion-controlled electron-transfer features operating on the modified electrode. The electron-hoping process, wherein, transfer of an electron from one redox-center to its neighboring site, is a likely reason for this observation.^58,59^

Based on the Laviron kinetics for the surface-confined redox system (peak-to-peak separation, Δ*E*_p_ < 200 mV), the heterogeneous electron-transfer rate constant, *k*_s_ was calculated.^59^ For that transfer coefficient value, *α* is first evaluated from the ratio of *E*_pa_ − *E*^f^/*E*_pc_ − *E*^f^*vs. n*Δ*E*_p_, wherein, *E*^f^ = formal potential (0.480 V *vs.* Ag/AgCl) and *n* = total number of electron involved (assuming it as 2), by substituting the data, Δ*E*_p_ = 52 mV at *v* = 200 mV s^−1^ (Fig. 2C), in Fig. 2 of the working plot of the Laviron model system,^60^ as 0.5. In next, the dimensionless parameter, *m* was calculated as 0.33 using the working Fig. 4 of the Laviron report.^60^ By substituting the above-mentioned parameters in the equation, *k*_s_ = *mnFv*/*RT*, wherein, *v* = scan rate and other parameters have its significance, *k*_s_ value was calculated as 5.1 s^−1^. This value is comparable with the value obtained from various surface-confined redox systems like 6.21 s^−1^ (GCE/CNF–Chit@Au_nano_)^47^ and 5.81 s^−1^ (BDD/thionine).^61^

**Fig. 3:**
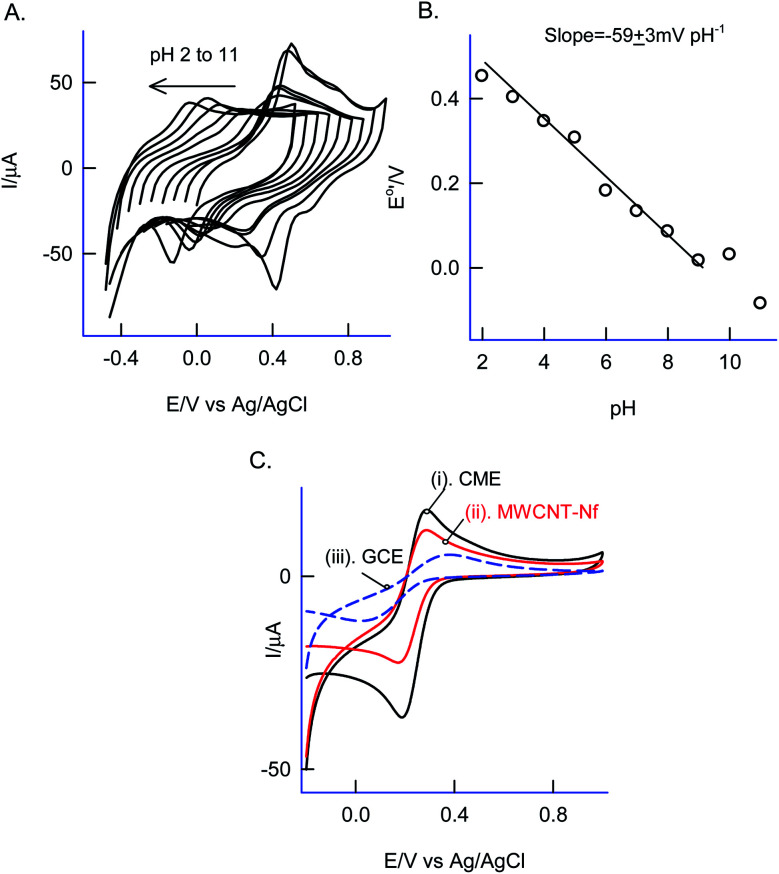
(A) Effect of solution pH on the CV response of GCE/Nf–MWCNT@Ph-OCH_3_-Redox at a fixed *v* = 50 mV s^−1^. (B) Plot of *E*^o^′ *vs.* solution pH. (C) CV responses of 5 mM Fe(CN)_6_ dissolved in 0.1 M KCl on GCE/Nf–MWCNT@PhOCH_3_-Redox (i), GCE/Nf–MWCNT (ii) and GCE (iii).

Effect of solution pH on the CV response of the GCE/Nf–MWCNT@PhOCH_3_-Redox was investigated as in Fig. 3A and B. A regular decrease in the redox peak potential upon increasing the solution pH upto pH 9, after that a plateau in the response was noticed. The obtained deviation in the pH, pH 9 is due to the acid-dissociation constant, p*K*_a_ of the redox-active composite system. A plot of apparent standard electrode potential, *E*^o^′ (*E*_pa_ + *E*_pc_/2) *vs.* pH yielded a linear line with a slope, ∂*E*^o^′/∂pH, −59 ± 3 mV dec^−1^ obeying the Nernstian type of *E*–pH relation with a theoretical slope value, −58 mV pH^−1^ corresponding an equal number of H^+^/e^−^ exchange reaction.

At this stage, it is difficult to predict the true species that is responsible for the redox reaction. In addition, since, the concentration of the immobilized redox species is expected to be in the nanogram level and exists as a composite with underlying carbon nanomaterials, isolation and characterization of the material could not lead to the real-molecular structure of the redox system. Besides, several physicochemical and electrochemical characterizations were performed to identify the nature of the redox peak formed in the chemically modified electrodes.

As a surface characterization, Fe^III/II^(CN)_6_^3−^ redox couple reaction was studied on GCE/Nf–MWCNT@PhOCH_3_-Redox in comparison with GCE and GCE/Nf–MWCNT in 0.1 M KCl solution. Fig. 3C is a comparative CV response at various modified electrodes. All the systems showed a qualitatively similar voltammetric feature. Indeed, in consideration with peak current values, the following is an obtained order of the modified electrodes: GCE/Nf–MWCNT@PhOCH_3_-Redox > GCE/Nf–MWCNT > GCE. The highest redox-peak current noticed with the GCE/Nf–MWCNT@PhOCH_3_-Redox indicates the superior electrical function of that system, which may be due to the existence of electro-active organic species formed within the matrix during the electrochemical procedure.

### 3.2 Influence of carbon nanostructure on the GCE/Nf–MWCNT@PhOCH_3_-Redox formation

**Fig. 4:**
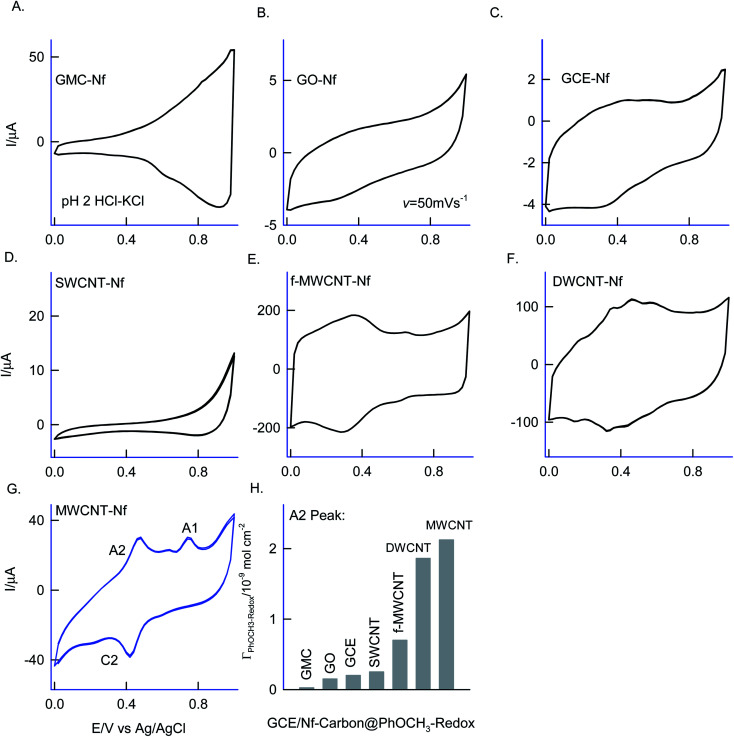
(A–G) Six continuous CV responses of various carbon@PhOCH_3_-Redox chemically modified electrodes prepared using 50 mM Ph-OCH_3_ + 6 mM H_2_O_2_ dissolved in 10 mL of pH 2 buffer at *v* = 50 mV s^−1^ and (H) comparison plot of surface excess (*Γ*) *vs.* various carbon matrixes for the PhOCH_3_-Redox formation. Graphitized mesoporous carbon = GMC, graphene oxide = GO, single-walled carbon nanotube = SWCNT, glassy carbon electrode = GCE, COOH-functionalized multiwalled carbon nanotube = f-MWCNT, double-walled carbon nanotube = DWCNT, multiwalled carbon nanotube = MWCNT. Nf = Nafion.

Different allotropes carbon nanomaterials such as SWCNT, GO, DWCNT, f-MWCNT, graphitized mesoporous carbon (GMC) after mixing with 0.5% Nafion were examined for the redox activity in pH 2 buffer + 6 mM H_2_O_2_ + 50 mM Ph-OCH_3_ mixture similar to ESI Fig. S1D† (Fig. 4). Each carbon material has its own-significance; SWCNT and DWCNT provide relatively less-denser CNT than that of the MWCNT; f-MWCNT offers an oxygen-rich surface functional group for molecular interaction; GO supports a planar graphitic structure with rich oxygen functional group and GMC provides a pores structure for the entrapment. By electrochemically interacting the PhOCH_3_ with the various carbon nanomaterials and from the results, the possible interaction mechanism between the electroactive species and carbon nanomaterial can be evaluated. Fig. 4A–G are typical CV responses of various carbon nanomaterial–Nafion modified GCEs prepared using PhOCH_3_ + H_2_O_2_ mixture. Based on the A2/C2 peak current signals, the order of PhOCH_3_-Redox formation is sequenced as GMC < GO < SWCNT < f-MWCNT ≪ DWCNT < MWCNT. Amongst all, DWCNT and MWCNT carbon nanomaterials modified electrodes provided the best redox peak with a quite prominent current response. Based on the results, following conclusions can be made: (i) dense graphitic structure is necessary for the strong π–π interaction between the aromatic units of PhOCH_3_ and sp^2^ carbon (graphitic) sites, (ii) oxygen-rich carbon nanomaterials are unfavorable for the electrochemical reaction. The surface oxygen functional group may trap some of the oxygenated intermediates of the PhOCH_3_-Redox through strong hydrogen bonding and hence it may hinder the electrochemical conversion. (iii) Confined and porous carbon structures are not suitable for PhOCH_3_ electrochemical reaction. There may be some kinetic restriction for the diffusion-controlled electrochemical oxidation of PhOCH_3_. In consideration of redox peak current and stability, the pristine MWCNT–Nf modified electrode has been chosen as an optimal system. (iv) The fraction of metal impurities in the pristine MWCNT have influenced the electrochemical PhOCH_3_-Redox formation. In our previous work, the ∼2 wt% iron impurity in the MWCNT played an important role in the immobilization of the lactoferrin enzyme on pristine MWCNT *via* a weak complexation between the iron-amino functional group and in turn for a facile electron-shuttling reaction.^62^ The iron content in the pristine-MWCNT was quantitatively characterized by XRD (after charing the MWCNT), TGA, ICP-MS and SEM-EDX techniques.^57^ Furthermore, an electro-Fenton-based hydroxylation of benzene to catechol and hydroquinone in acidic solution was also observed.^27^ Meanwhile in the literature, homogeneous condition-based oxidation of aryl methoxy and heteroatom-containing aromatic units to respective aryl hydroxy derivatives at high oxidation potential, >1 V *vs.* Ag/AgCl^63,64^ were reported. In this work, we believed that the {HOO–Fe^*n*+^/O(CH_3_)-Ph, *n* = 0–2} complex is formed with the aid of π–π interaction between benzene-ring of PhOCH_3_ and sp^2^ carbon of MWCNT, similar to the mechanism proposed by Hamilton and Friedman,^4^ helped in the overall heterogeneous-molecular transfer reaction (Scheme 1). This is the reason why metal impurity-free carbon matrixes such as CNF, GO and GCE have failed to show any such unique electrochemical conversion.

**Scheme 2:**
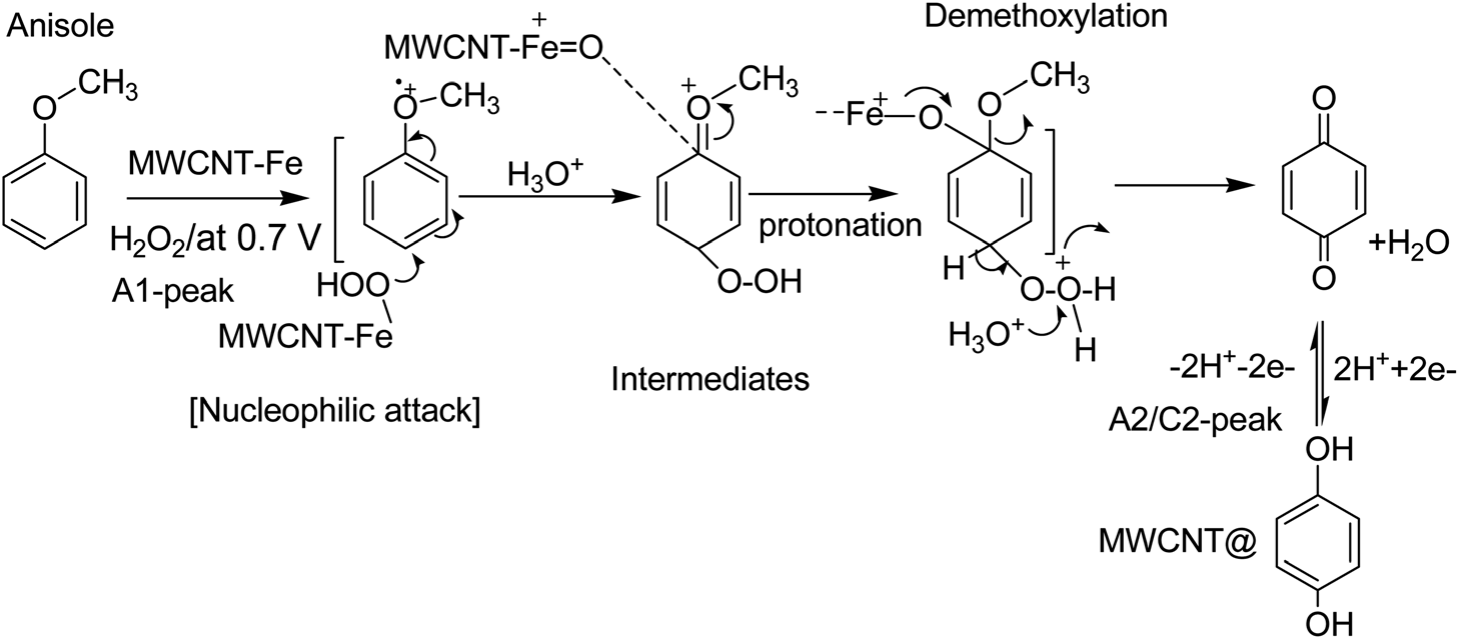
Illustration of the reaction mechanism for the pristine-MWCNT and H_2_O_2_ assisted molecular conversion of anisole to surface-confined hydroquinone, MWCNT@HQ. Note: HQ = PhOCH_3_-Redox.

To understand the role of Nafion on the Nf–MWCNT@PhOCH_3_-Redox formation, varying amounts, 0.5–5% were combined with MWCNT and investigated further. ESI Fig. S2† is a typical CV response of GCE/Nf (0.5–5%)–MWCNT@PhOCH_3_-Redox in a blank pH 2 KCl–HCl solution. Up to 1% Nafion-modified system, an increase in the A2/C2 redox peak current, after that a decline in the current response, was observed. Indeed, in consideration of the peak current stability and reproducibility, a 0.5%-Nafion solution modified system has provided the best voltammetric response. We believed that at an optimal condition, the Nf–MWCNT composite electrode provided an acidic environment (due to the –SO_3_^−^·H^+^) to the modified electrode, which is a favorable condition for the existence of the iron oxide (Fe_2_O_3_) as an anion species (Fe^2+^O_*x*_/Fe^1+^O_*x*_ or –^*n*+^Fe=O) and further for weak complexation-complex with carbon–oxygen-site of PhOCH_3_ (demethoxylation) and H_2_O_2_ (OOH) (Scheme 2). Higher the Nafion loading may result in a large hydrophilic layer and hindrance for the π–π interaction of the reactant on the MWCNT surface. Similarly, the effect of H_2_O_2_ concentration on the formation of PhOCH_3_-Redox was studied as in ESI Fig. S3,† showing a stable voltammetry response at 6 mM of H_2_O_2_. It is like that at this concentration there will be an optimal formation of {HOO–Fe–PhOCH_3_}@MWCNT complex system.

Based on our previously published works on the electrochemical hydroxylation phenolic functional group^65,66^ and benzene^27^ to respective quinone like derivative/s at pristine MWCNT, the following four types of mechanisms are proposed: case-i, direct oxidation of phenolic functional group to phenolic radical intermediate at about 0.5 V *vs.* Ag/AgCl (*i.e.*, 0.7 V *vs.* Ag/AgCl in pH 2; A1 peak) followed by hydroxylation reaction in an aqueous medium; case-ii, dissolved oxygen reduction reaction (ORR) to hydrogen peroxide intermediate and hydroxylation of the MWCNT surface-bound organic molecule, *via* π–π interaction, at −0.4 V *vs.* Ag/AgCl in pH 7 PBS; case-iii, formation of peroxy/hydroxyl radical intermediate upon water oxidation reaction at 1.2 V *vs.* Ag/AgCl in pH 7 PBS and its assistance on the electrochemical oxidation of surface-confined organic molecules to the redox-active system and case-iv, electro-Fenton type hydroxylation reaction, wherein, electrochemical reaction of intrinsic Fe containing carbon nanomaterial with benzene in presence of deliberately added H_2_O_2_ in acidic medium for the formation of mixture of catechol and HQ. Meanwhile, hydroxylation procedure based on Hamilton and Friedman approach, wherein, {H_2_O_2_–Fe^2+^–dihydroxybenzene} complex like catalytic system for the nucleophilic based hydroxylation reaction of phenolic precursors (case-v) is also accounted for PhOCH_3_-Redox formation. By performing the potential window effect, a possible mechanism for the Nf–MWCNT–PhOCH_3_/H_2_O_2_-electrochemical oxidation reaction can be predicted.

### 3.3 Effect of the potential window on the preparation of the modified electrode

**Fig. 5:**
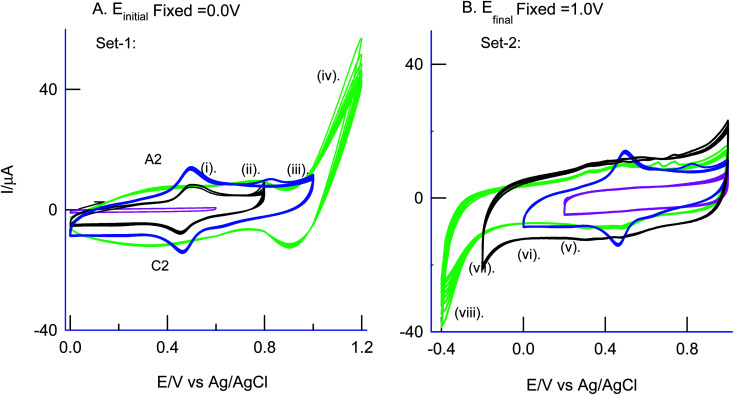
Effect of bias potential on CV response of GCE/Nf–MWCNT (freshly prepared electrodes) with 50 mM PhOCH_3_ + 6 mM H_2_O_2_ in pH 2 KCl–HCl buffer at *v* = 50 mV s^−1^ (A) CV experiments with a fixed starting/initial potential of 0.0 V and varying switching final potential of (i) 0.6 V, (ii) 0.8 V, (iii) 1.0 V and (iv) 1.2 V *vs.* Ag/AgCl. (B) CV experiments with a fixed final potential of 1.0 V and varying initial potentials from (v) −0.4 V, (vi) −0.2 V, (vii) 0.0 V and (viii) 0.2 V *vs.* Ag/AgCl.

**Fig. 6:**
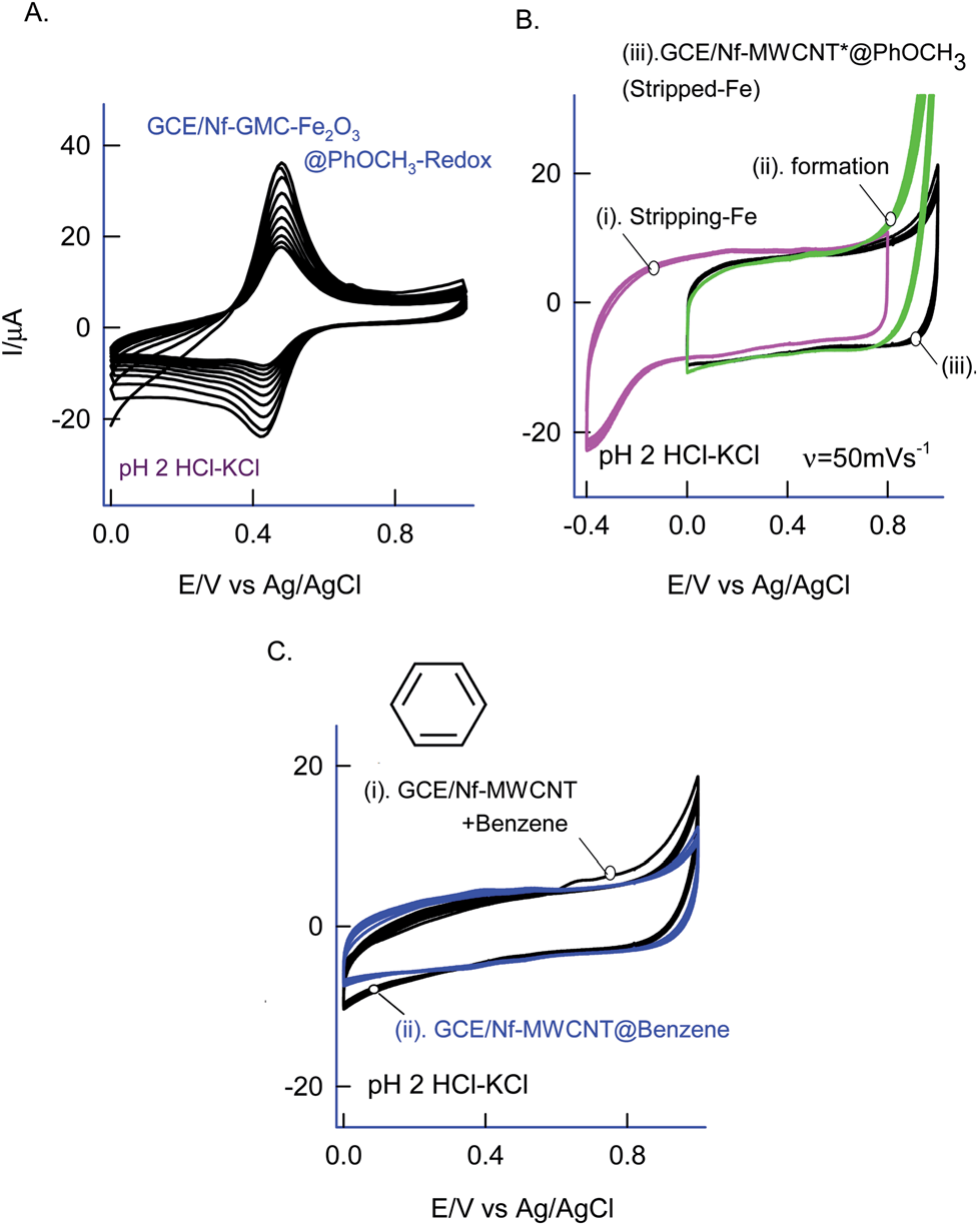
(A) Twenty CV segments of GCE/Nf–GMC{Fe_2_O_3_}@PhOCH_3_-Redox prepared using Fe_2_O_3_ externally added Nf–GMC modified electrode with 50 mM PhOCH_3_ + 6 mM H_2_O_2_ in 10 mL of pH 2 at *v* = 50 mV s^−1^. (B) CV response of GCE/Nf–MWCNT in pH 2 KCl–HCl for the electrochemical assisted stripping of iron oxide impurities (i), its responses with 50 mM PhOCH_3_ + 6 mM H_2_O_2_ in pH 2 KCl–HCl (ii) and its PhOCH_3_-Redox chemically modified electrode (iii). (C) CV response of GCE/Nf–MWCNT with 50 mM benzene + 6 mM H_2_O_2_ (curve (i)) and its blank transfer chemically modified electrode (curve (ii)) in pH 2 KCl–HCl solution.

As in Fig. 5A and B, two sets of CV experiments were carried out in which, in the first set of experiments (Set-1), the starting potential of the GCE/Nf–MWCNT is fixed as 0 V *vs.* Ag/AgCl and varied the final potential as 0.6 (i), 0.8 (ii), 1.0 (iii) and 1.2 V *vs.* Ag/AgCl (iv). Similarly, in the 2^nd^ set of experiments (Set-2), end-potential is fixed as 1.0 V *vs.* Ag/AgCl and varied the starting potential as 0.2 (v), 0.0 (vi), −0.2 (vii) and −0.4 V *vs.* Ag/AgCl (viii). The potential cycling experiment carried out at a window, 0 to 1 V *vs.* Ag/AgCl (Set-1 (iii) and Set-2 (vi)) showed an optimal response for the A2/C2 redox peak formation. Sweeping the potentials to more positive and negative sides, *i.e.*, 1.2 and −0.4 V independently, wherein OER and ORR occurred respectively, were resulted in a poor faradaic response. These observations rolled-out the case-ii and case-iii types of reaction mechanisms. Ultimately, direct oxidation of the PhOCH_3_ at about 0.70 V *vs.* Ag/AgCl (A1) followed by nucleophilic addition of ˙OOH radical species from Fe–OOH complex precursor is likely the key step for the electrochemical reaction in this work. In connection to this, homolytic O–O bond cleavage and formation of Fe^III^–OOH complex have been reported for some organic substrates oxidation reaction by nonheme iron catalysts with H_2_O_2_.^67^ To support the overall mechanism proposed in this work, metal impurity-free carbon nanomaterials like graphitized mesoporous carbon (GMC) and carbon nanofiber (CNF) are tested for the PhOCH_3_-electrochemical reaction, after being deliberately modified with Nafion–Fe_2_O_3_ mixture on the surface (Fig. 6A and ESI Fig. S4B†). A qualitatively similar voltammetric A2/C2 peak was noticed supporting the specific role of iron impurity and graphitic structure for the PhOCH_3_-electrochemical reaction. In continuation, iron-impurities removed MWCNT, which is obtained potential cycling of the MWCNT modified electrode in mild acid condition as per previous literature report,^27,68^ was tested for the PhOCH_3_-Redox formation but failed to show any A2/C2 peak formation authenticating the specific role of iron in the electrochemical reaction (Fig. 6B). Another control for GCE–Nf(Fe_2_O_3_)@PhOCH_3_-Redox with GCE–Nf@PhOCH_3_-Redox was studied as in ESI Fig. S4A.† The appearance of a feeble peak like a response for the GCE–Nf(Fe_2_O_3_)@PhOCH_3_-Redox only. In addition, a control experiment based on Fe_2_O_3_ deliberately added GCE/Nf–MWCNT system was tested for the PhOCH_3_-Redox formation showing the specific formation of A2/C2 redox peak (ESI Fig. S4C†). These results confirm the important role of intrinsic iron in the conversion of in-active anisole to the electro-active molecule, PhOCH_3_-Redox.

Based on the above results and from the observation of the intrinsic iron-based MWCNT on the specific electrochemical reaction, a possible mechanism for the PhOCH_3_ is sketched in Schemes 1A–C and 2. In that scheme, electrochemical oxidation of ether-site of the surface-confined PhOCH_3_ to respective cationic radical species (A1 peak) followed by complexation reaction with Fe^III^–OOH species for the simultaneous formation surface-confined HQ is proposed. Such a unique molecular transfer reaction is differing distinctly from the electro-Fenton based hydroxylation reaction (case iv). To verify it, under an identical experimental condition, GCE/Nf–MWCNT electrode is subjected to the benzene-electrochemical reaction in presence of H_2_O_2_ in pH 2 KCl–HCl solution (Fig. 6C), similar to the previous work on electro-Fenton reaction using pristine-MWCNT.^27^ Interestingly, there is no observation of specific redox peak corresponding to hydrated benzene, *i.e.*, CA and HQ, rolling-out operating of the Fenton-based reaction mechanism in this work.

### 3.4 Physicochemical characterization of MWCNT@PhOCH_3_-Redox

**Fig. 7:**
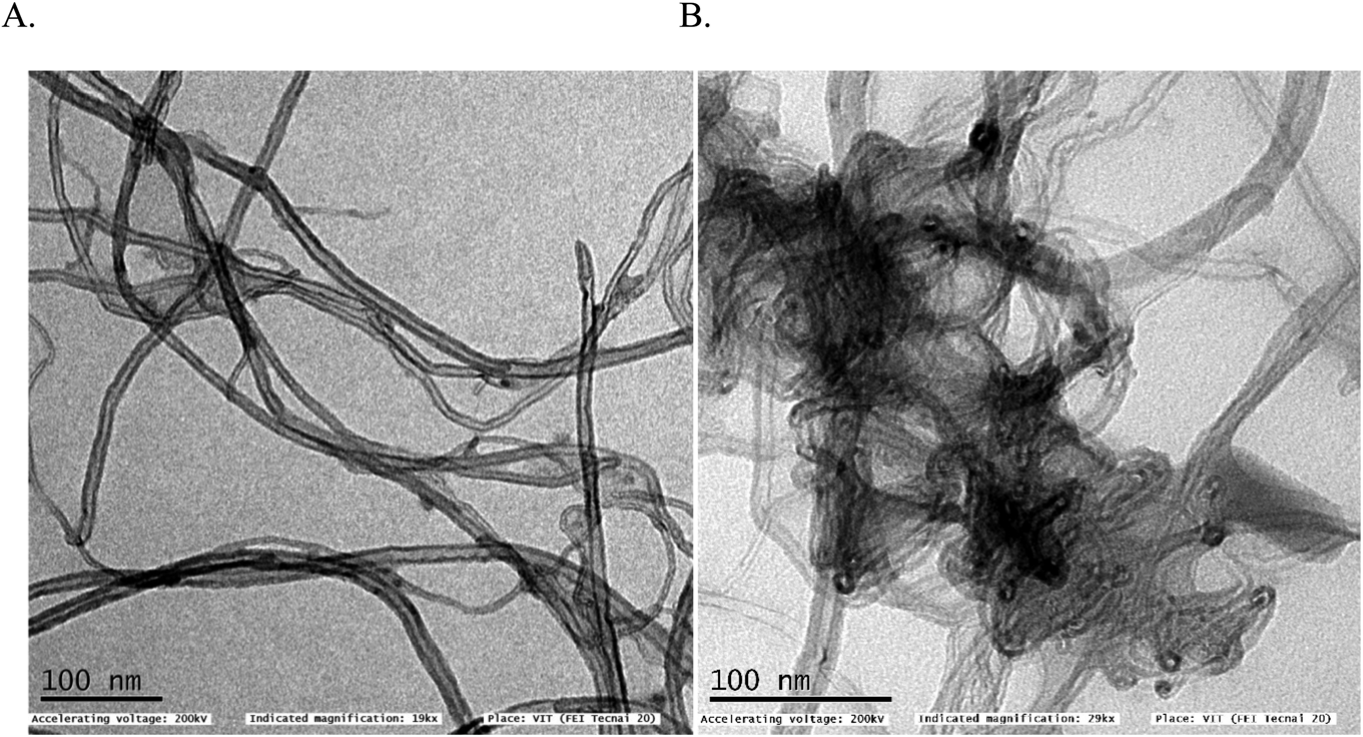
TEM images of (A) unmodified MWCNT and (B) MWCNT@PhOCH_3_-Redox systems.

**Fig. 8:**
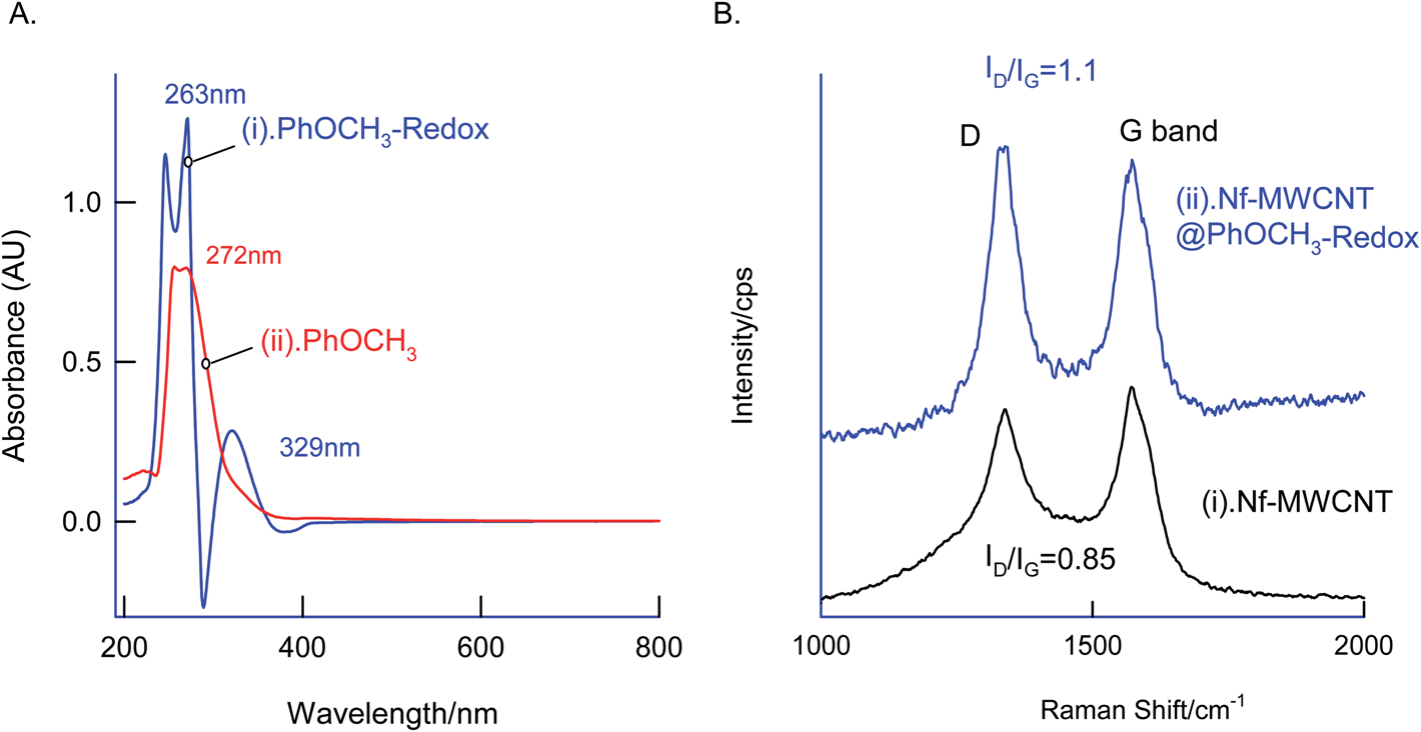
Physicochemical characterization of the Nf–MWCNT@PhOCH_3_-Redox with control samples using (A) Raman spectra and (B) UV-Vis spectroscopic techniques. For UV-Vis analysis of PhOCH_3_-Redox, an ethanol extract GCE/Nf–MWCNT@PhOCH_3_-Redox followed by column filtered sample was used. Screen-printed electrode modified was used for Raman spectroscopic analysis.

In first, TEM technique was used for the characterization of MWCNT (A) and MWCNT@PhOCH_3_-Redox (B) comparatively as in Fig. 7. For practical convenience, the Nafion layer was not included in this system. Unmodified MWCNT showed an irregular arrangement of a hollow nano-tubular structure. But, after the electrochemical modification, a cloudy nanotube structural morphology was noticed. As first-hand information, the result provides detail about a strong immobilization of the redox-active organic moiety on the MWCNT surface, probably by π–π transaction. To identify the nature of the molecular system, UV-Vis spectroscopy examination of an ethanolic extract of MWCNT@PhOCH_3_-Redox was investigated. Fig. 8 is a typical UV-Vis response of unreacted PhOCH_3_, showing a *λ*_max_ at 270 ± 2 nm, whereas, the PhOCH-Redox sample yielded signal at *λ*_max_ = 265 ± 2 and 330 ± 2 nm. The shift in the wavelength accounts for changes in the molecular structure, possibly hydroquinone (Ph-OCH_3_-Redox = HQ) like product formation.^69^ This information ascribes for a new organic product–MWCNT composite formation in this work. In further, Raman spectroscopic analysis was carried out to find out the qualitative information about the interaction of the organic moiety with the underlying MWCNT. Fig. 8B is a comparative response of Nf–MWCNT and Nf–MWCNT@PhOCH_3_-Redox systems. Specific peaks at 1350 and 1550 cm^−1^ corresponding to the disordered (D band) and ordered graphitic (G band) signals of the modified system were noticed. The intensity ratio of *I*_D_/*I*_G_ is taken as a sensitive parameter to compare their molecular structure. Calculated *I*_D_/*I*_G_ ratio increased from 0.85 to 1.1 indicating that part of the sp^2^ (G band) is converted to sp^3^ (bonding) sites. Presumably, there is a formation of dihydroxybenzene derivative like a hydroquinone molecule upon the electrochemical reaction.

**Fig. 9:**
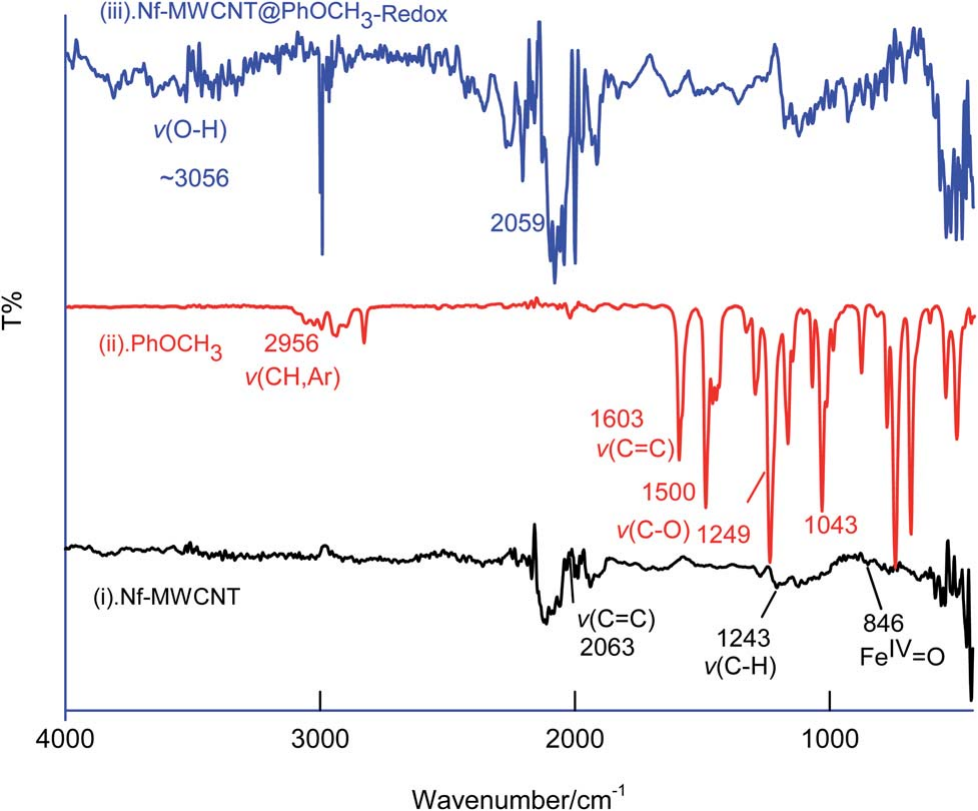
Comparative FTIR characterization responses of Nf–MWCNT (curve (i)), PhOCH_3_ (curve (ii)), Nf–MWCNT@PhOCH_3_-Redox (curve (iii)).

FT-IR spectroscopic studies were further carried out to reveal functional groups. As can be seen in Fig. 9, presence of 3056 cm^−1^*ν*(–O–H; hydroxy), 2059 cm^−1^*ν*(–C–H; bending), 1124 cm^−1^*ν*(C=C; aromatic), 790 cm^−1^*ν*(Fe^IV^=O) signals were noticed with GCE/Nf–MWCNT@PhOCH_3_-Redox system, whereas, GCE/MWCNT showed signals at 2063 cm^−1^*ν*(C=C), 1243 cm^−1^*ν*(C–H; bending), 846 cm^−1^*ν*(Fe^IV^=O). Similarly, the reaction molecule, Ph-OCH_3_ was also subjected for the studies yielding IR peaks at 2956 cm^−1^*ν*(CH; aromatic), 1603 cm^−1^ and 1500 cm^−1^*ν*(C=C; aromatic; benzene), 1249 cm^−1^ and 1043 cm^−1^*ν*(C–O; ester). The above information reveals that the ester bond at 1043 cm^−1^ with the formation of a new peak at >3000 cm^−1^ due to hydroxy-functionalized moiety formation. This observation was similar to our previous report for the benzene electro-Fenton reaction to dihydroxybenzene products,^27^ but with different reaction-mechanism in this work. These observations indicated that HQ or its derivative like a product is formed on the Nf–MWCNT surface and showed the A2/C2 redox peak.

**Fig. 10:**
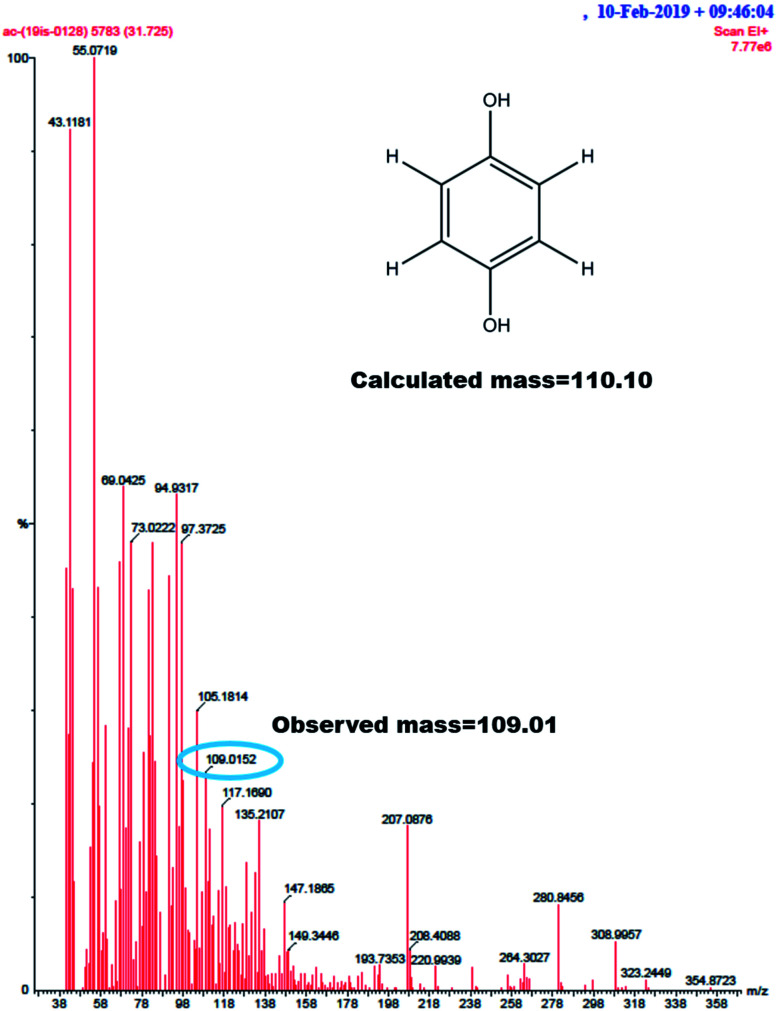
GC-MS of PhOCH_3_-Redox showing *m*/*z* = 109.01, Ph-(OH)_2_ (hydroquinone; M–H peak) (calculated, 110.10).

**Fig. 11:**
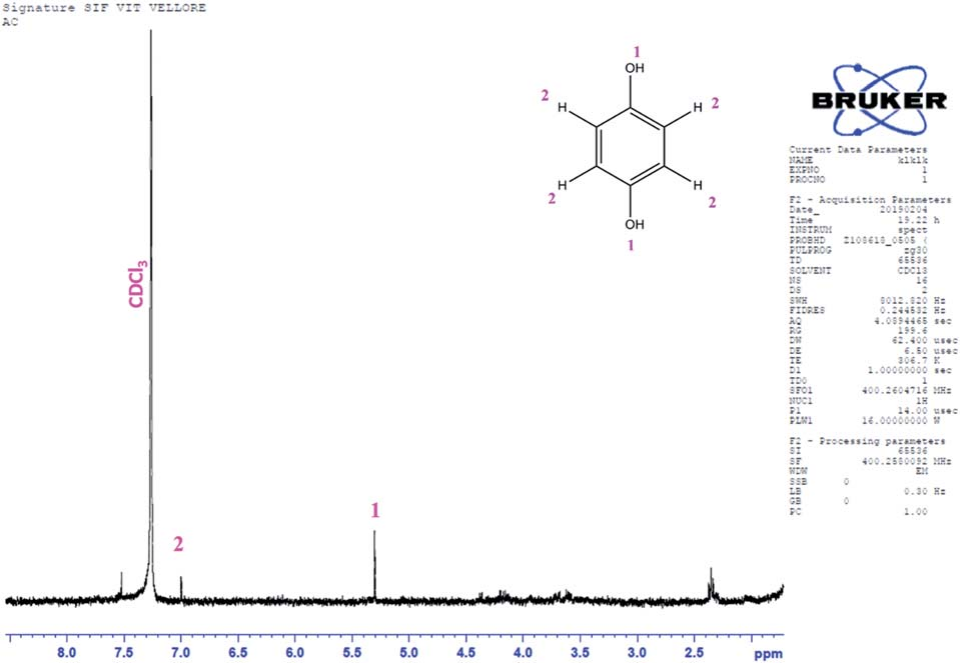
NMR spectra of the Ph-OCH_3_-Redox extract obtained after cycling voltammetry of GCE/Nf–MWCNT with Ph-OCH_3_ dissolved in ethanol followed by column filtration. Extract was dried and NMR analysis was performed using CDCl_3_ as a solvent. Insets are typical illustrations of respective organic compounds and its positions.

GC-MS characterization was performed to understand the product of A2/C2 redox peak. The ethanolic extract of the PhOCH_3_-Redox after purification in column chromatography was analyzed. Fig. 10 is a typical GC-MS response of the product showing a specific *m*/*z* peak at 109.01 (CAT or HQ; calculated 110.10). From the collective results, it is confirmed that CA or HQ is the sole product of the electrochemical oxidative reaction of PhOCH_3_ system while control for PhOCH_3_ is in ESI Fig. S5A.† To further confirm the molecular structure, NMR experiment was carried out for both isolated product and reactant molecule using CDCl_3_ solvent as shown in Fig. 11. The presence of 7.3 ppm (multiplet; 1H; at C2), 6.8 ppm (multiplet; 1H; at C1 and C3) and 3.7 ppm (singlet; –OCH_3_ at C4) confirms phenyl methyl ether, whereas, peaks at 6.9 ppm (singlet; 1H; at C2) and 5.3 ppm (singlet; OH; at C1) evidenced hydroquinone as an organic moiety^70^ being formed after PhOCH_3_ electrochemical reaction. Control NMR for PhOCH_3_ is provided in ESI Fig. S5B.† Note that such a unique molecular transformation is the first time reported in this work. At this point onwards the GCE/MWCNT–Nf@PhOCH_3_-Redox is redesignated as GCE/Nf–MWCNT@HQ, wherein, PhOCH_3_-Redox = HQ. Note that after the electrochemical preparation procedure, the modified electrode is washed with double distilled water and performed the CV experiment. It is likely that upon the washing procedure the loosely bounded, organic matters were stripped out from the surface and leaving the electro-active active product, HQ on the interface. This procedure is quite different from the conventional electro-organic synthetic procedure, where, the bulk reactant was involved in the electro-synthetic procedure. Regarding the quantitative efficiency of the conversion, since the electrochemical reaction is based on a homogeneous to surface-confined electrochemical process on the tiny electrode, the exact efficiency detail is difficult to measure.

The stability was tested by measuring the peak current values at a continuous cycling condition which showed a RSD value 2.1% (twenty cycles). In addition, if the modified electrode is stored for a week time in a closed room temperature, *T* = 28 ± 2 °C, there is no marked alteration in the peak current and peak potential noticed highlighting the appreciable stability of the modified electrode.

### 3.5 Electrocatalytic reduction of Cr(vi) by GCE/Nf–MWCNT@HQ

**Fig. 12:**
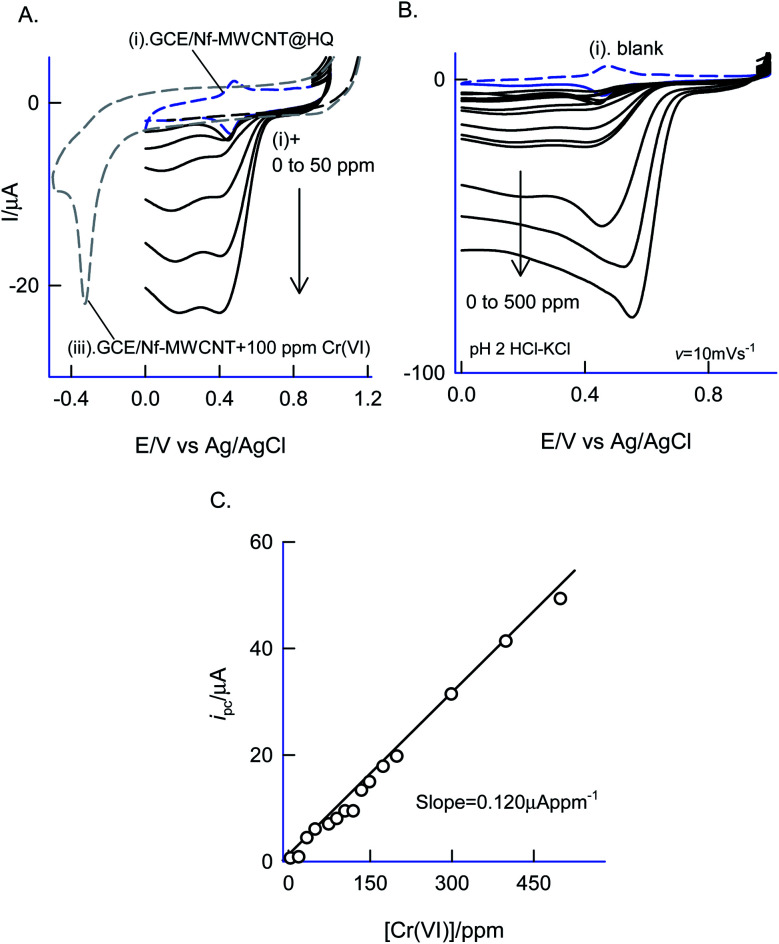
(A and B) CV responses of GCE/Nf–MWCNT@HQ (Ph-OCH_3_-Redox) without (curve (i)) and with various concentrations of Cr_2_O_7_^2−^ (curve (ii)) at *v* = 10 mV s^−1^ in pH 2 KCl–HCl buffer. Curve (iii) is a control CV response of GCE/Nf–MWCNT with 100 ppm of Cr_2_O_7_^2−^ in pH 2 KCl–HCl solution. (C) Calibration plot of (C) *i*_pc_*vs.* Cr_2_O_7_^2−^ concentration.

Fig. 12 is a CV experiment for electrochemical reduction of different chromium(vi) concentrations, 5 to 500 ppm on GCE/Nf–MWCNT@HQ in pH 2 HCl–KCl (Fig. 12A and B). A specific electrochemical reduction peak potential, *E*_pc_ at 0.35 ± 0.05 V *vs.* Ag/AgCl, near to the A2/C2 redox peak due to HQ ⇔ Q + 2H^+^ + 2e^−^ redox couple exists, was noticed. As a control experiment, GCE/Nf–MWCNT was subjected to 100 ppm Cr(vi) reduction reaction yielding a feeble peak at *E*_pc_ ∼ −0.4 V *vs.* Ag/AgCl (Fig. 12, curve (i)), which is about 800 mV reduction in the over-potential value and about two times lower in the peak current signal. In continuation, GCE/MWCNT has also been subjected to Cr(vi) reduction reaction showing an absence of any such electrochemical reduction response (ESI Fig. S6†). These observations denote the electrocatalytic Cr(vi) reduction performance of the chemically modified electrode. A plot of *i*_pa_/μA *vs.* concentration of Cr(vi)/ppm yielded a wide linear calibration range, 5–500 ppm Cr(vi) at a scan rate of *v* = 10 mV s^−1^ with a current sensitivity value, 0.120 μA ppm^−1^ (Fig. 12C).^71^ Based on the results, a plausible mechanism for the electrocatalytic reduction of Cr(vi) ion proposed that the reduced form of quinone, hydroquinone chemically reduce the Cr(vi) to Cr(iii) followed by transformation as an oxidized organic species, quinone. The HQ is regenerated-back at an operating potential, 0.4 V *vs.* Ag/AgCl on the modified electrode surface as sketched in Scheme 1C. The HQ* commercial sample stability was examined at a potential window similar to the optimal system, but showing a poor voltammetric response as shown in the ESI Fig. S7.† This observation ruled out the use of commercial samples for the chemically modified electrode preparation and electrocatalytic application.

**Fig. 13:**
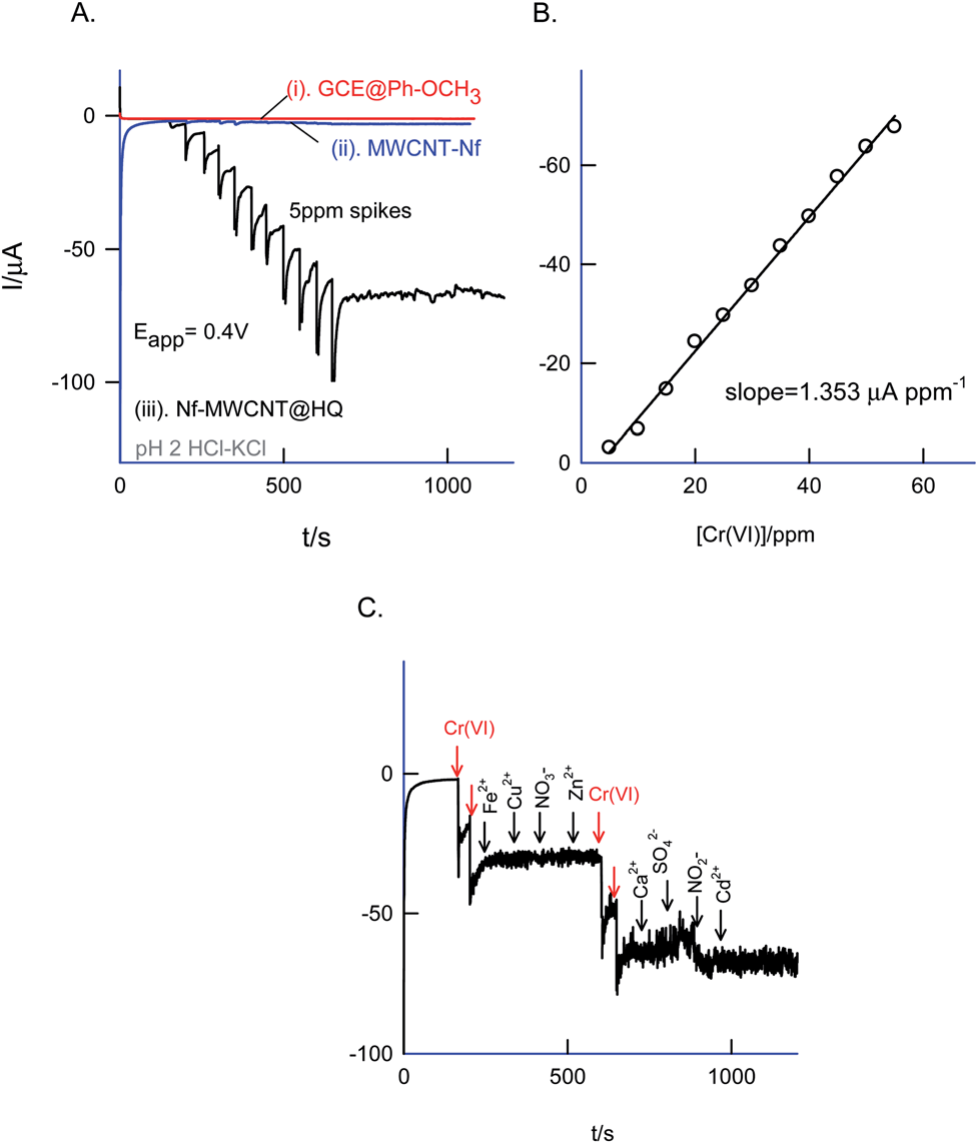
(A) Comparative amperometric *i*–*t* responses of GCE@Ph-OCH_3_ (curve (i)), GCE/Nf–MWCNT (curve (ii)) and GCE/Nf–MWCNT@HQ (curve (iii)) for successive additions of 5 ppm Cr(vi) at *E*_app_ = 0.4 V *vs.* AgCl in 10 mL pH 2 KCl–HCl buffer (hydrodynamic condition). (B) Typical calibration plot. (C) Amperometric *i*–*t* response of the GCE/Nf–MWCNT@HQ with successive additions of different interfering chemicals 10 ppm Cr(vi) followed by 10 ppm Cr(vi), iron(iii), copper(ii), nitrate, zinc(ii), sulfate, calcium(ii), nitrite, cadmium(ii).

The sensor system was further extended to amperometric *i*–*t* detection by spiking dilute Cr(vi) solutions into a blank pH 2 KCl–HCl at an applied potential, *E*_app_ = 0.4 V *vs.* Ag/AgCl under hydrodynamic condition. The step-wise amperometric *i*–*t* response measured for each 5 ppm addition of dichromate in pH 2 solution after every 50 seconds up to twelve such additions were displayed in Fig. 13A. Control experiments relating to GCE@Ph-OCH_3_ and GCE/Nf–MWCNT were performed with the Cr(vi) additions as in Fig. 13A. It is obvious to see a marked sensing response for the electrocatalytic reduction of Cr(vi) by the HQ modified electrode, whereas, the control electrodes failed to show any such unique response. The RSD, sensitivity and detection limit were calculated using respective calibration as 2.4%, 1.3 μA ppm^−1^ and 230 ppb respectively (Fig. 13B) which is comparable with other reports.^72–74^

**Fig. 14:**
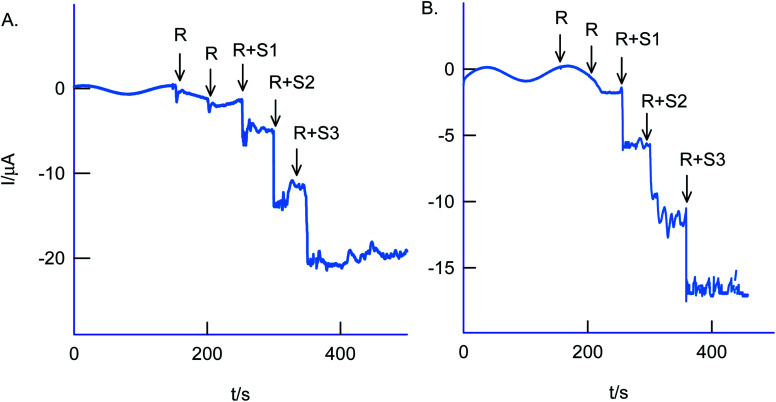
Amperometric *i*–*t* based real sample analysis of Cr(vi) species in two industrial effluents samples using GCE/Nf–MWCNT@HQ modified electrode in pH 2 KCl–HCl solution. A four-time diluted test sample with pH 2 KCl–HCl was subjected for the analysis. *E*_app_ = 0.4 V *vs.* Ag/AgCl. R = real sample, R + S1–S3 = real + three different standard Cr(vi) concentration numbers, 1–3.

The selectivity of the chemically modified electrode was tested with various referencing systems like copper, ferrous, nitrite, zinc, sulfate, calcium, nitrate and cadmium as in Fig. 13C, but there were no specific current signals noticed highlighting the specificity of the GCE/Nf–MWCNT@HQ for the Cr(vi) sensing. To validate the approach, a couple of tannery industry water samples were subjected to Cr(vi) sensing by standard addition approach as in Fig. 14. The standard addition approach, in which standard concentration of Cr(vi) ion along with real-sample performed, is well-recommended in the analytical chemistry. Note that the atomic absorption spectroscopic (AAS) based analytical approach will provide the detail of total Cr content, not of Cr(vi) speciation. The electroanalytical parameter for the assay was displayed in Table 2. Real sample Cr(vi) concentration of 0.3 ppm and 0.2 ppm detected in the pollute water sample. Calculated recovery values are 100 ± 5% ascribing the suitability of the present technique for various Cr(vi) real sample analysis. Since the new chemically modified electrode showed a stable and efficient electrocatalytic response, it can be extended as an electrochemical detector in flow injection analysis coupled electrochemical sensing platform.

**Table 2:** Electrochemical sensing of H_2_O_2_ in various real samples using GCE/MWCNT@HQ modified electrode. R – real sample (1 mL), R + S1–S3, real sample + standard concentrations^a^

Real sample	Analysis	Detected (μM)	Spike (μM)	Recovery (%)
(1) Sample #1	R	0.3	—	—
	R + S1	3.3	3	103.4
	R + S2	5.7	6	96.9
	R + S3	9.1	9	100.4
(2) Sample #2	R	0.2	—	—
	R + S1	3.2	3	102.7
	R + S2	5.8	6	98.8
	R + S3	9.0	9	100.0

a R = real sample; S = standard solution; dilution factor = 2 : 8 *i.e.* (real sample : pH 2 HCl–KCl); R + S1 = real sample + standard Cr(vi) solution with addition 1.

## 4. Conclusions

Electrochemical oxidation of anisole on the Nafion–pristine MWCNT composite modified electrode by continuous potential cycling in pH 2 KCl–HCl has been performed. A well-defined surface-confined redox peak at *E*^o^′ = 0.45 V *vs.* Ag/AgCl (A2/C2) with a surface-excess value 2.1 × 10^−9^ mol cm^−2^ was obtained. No such specific A2/C2-redox peak observation was noticed when other carbon nanomaterials such as SWCNT, GO, GMC on GCE was used as an underlying surface. To find out the true organic redox species responsible for the redox peak formation, various physicochemical characterizations using TEM, UV-Vis, Raman, FTIR, NMR, GC-MS and specific electrochemical characterization were performed. From the collective results, it has been revealed that the electro-inactive PhOCH_3_ gets oxidized as respective oxygen radical species at the positive potential at *E*_pa_ = 0.7 V *vs.* Ag/AgCl (A1) and involved in {HOOH–Fe–O(CH_3_)Ph} complexation on MWCNT surface (in presence of H_2_O_2_) followed by *in situ* demethoxylation and hydroxylation to HQ-product formation (Schemes 1 and 2). The HQ modified electrode showed an efficient electrocatalytic reduction signal for Cr(vi) ion in pH 2 KCl–HCl solution. The calibration plot was linear in a range 5–500 ppm of Cr(vi) by cyclic voltammetric and amperometric sensing techniques. Investigated interference effect by amperometric *i*–*t* showed the absence of any signal with various common metal ion systems like copper, ferrous, nitrite, zinc, sulfate, calcium, nitrate and cadmium. As a proof of concept, specific detection of Cr(vi) present in the tannery industry water waste has been demonstrated with a recovery value 100 ± 5%.

## Conflicts of interest

The authors declare no competing financial interest.

## Supplementary Material

RA-011-D0RA10370E-s001
