# Supplementary material for: In situ electro-organic synthesis of hydroquinone using anisole on MWCNT/Nafion modified electrode surface and its heterogeneous electrocatalytic reduction of toxic Cr(vi) species
Source: RSC Adv. 2021 Jan 20;11(7):4062–76. doi: 10.1039/d0ra10370e (PMC8694528; doi:10.1039/d0ra10370e)
Supplement: RA-011-D0RA10370E-s001 [file RA-011-D0RA10370E-s001.pdf]

Electronic Supplementary Material (ESI) for RSC Advances.
This journal is © The Royal Society of Chemistry 2021

Supporting Information

In-situ Electro-Organic Synthesis of Hydroquinone Using Anisole on MWCNT/Nafion Modified Electrode Surface and Its Heterogenous Electrocatalytic Reduction of Toxic Cr(VI) Species

Mansi Gandhi,[a,b] Desikan Rajagopal*[b] and Annamalai Senthil Kumar*[a,b,c]

[a]*Nano and Bioelectrochemistry Research Laboratory, Department of Chemistry, School of Advanced Sciences, Vellore Institute of Technology University, Vellore-632014, India*

[b]*Department of Chemistry, School of Advanced Sciences, Vellore Institute of Technology University, Vellore-632014, India*

[c]*Carbon dioxide Research and Green Technology Centre, Vellore Institute of Technology University, Vellore-632014, Tamil Nadu, India*

*One of the corresponding Author's email: askumarchem@yahoo.com; askumar@vit.ac.in

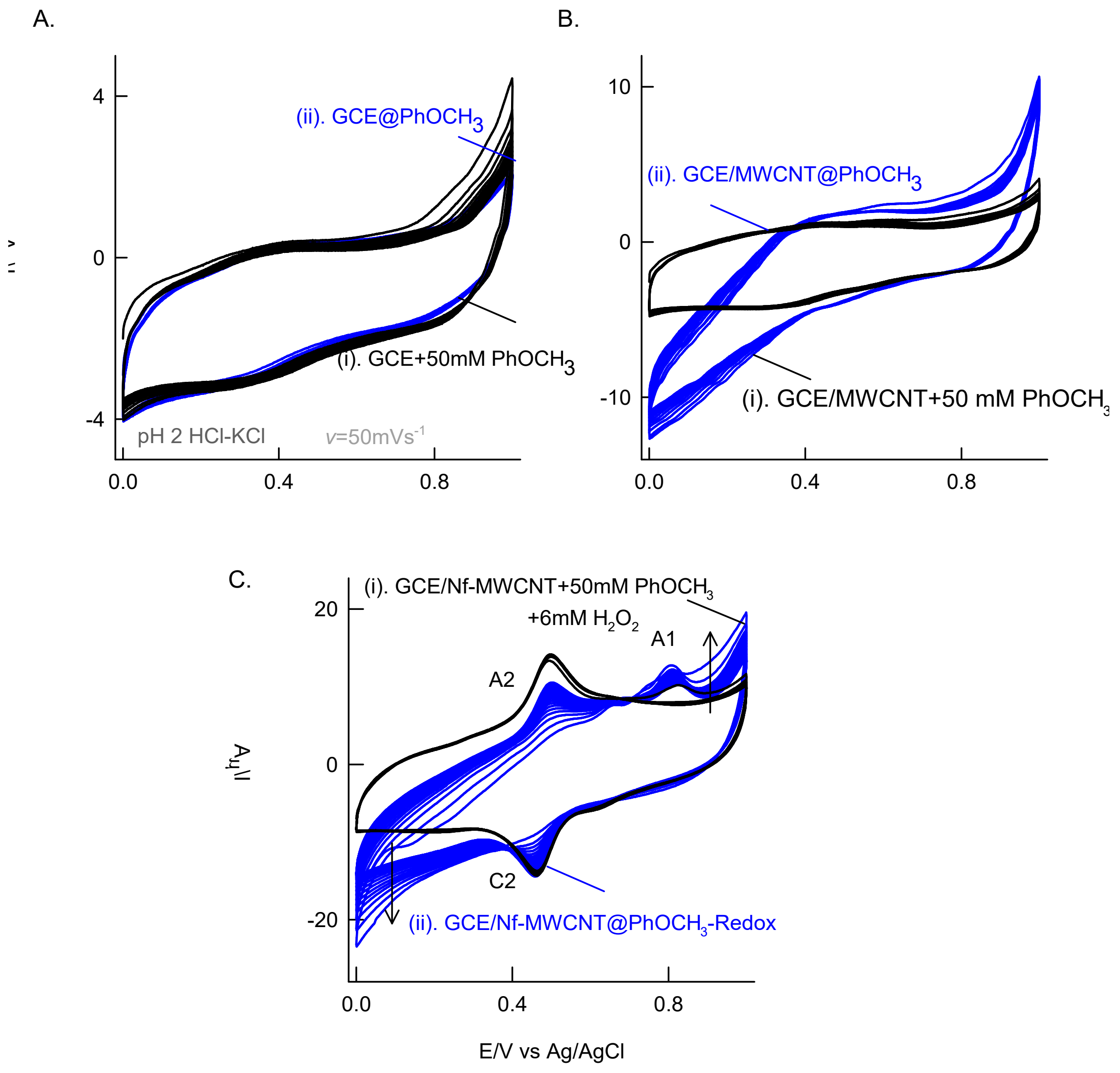

Supplemnetary Fig. S1. Twenty continuous CV segments of (A) GCE and (B) GCE/MWCNT in 50mM $PhOCH_3$ dissolved in 10 mL pH 2 KCl-HCl buffer (curve (i)) and its medium transferred CV responses (curve (ii)). CV responses of (C) GCE/Nf-MWCNT in a mixture of 50mM $PhOCH_3$ + 6mM H_2O_2 dissolved in 10mL of pH 2 KCl-HCl buffer (curve (i)) and its medium (blank pH 2) transferred response (curve (ii)) for at ν=50mVs^{-1}. Note: Nf=Nafion.

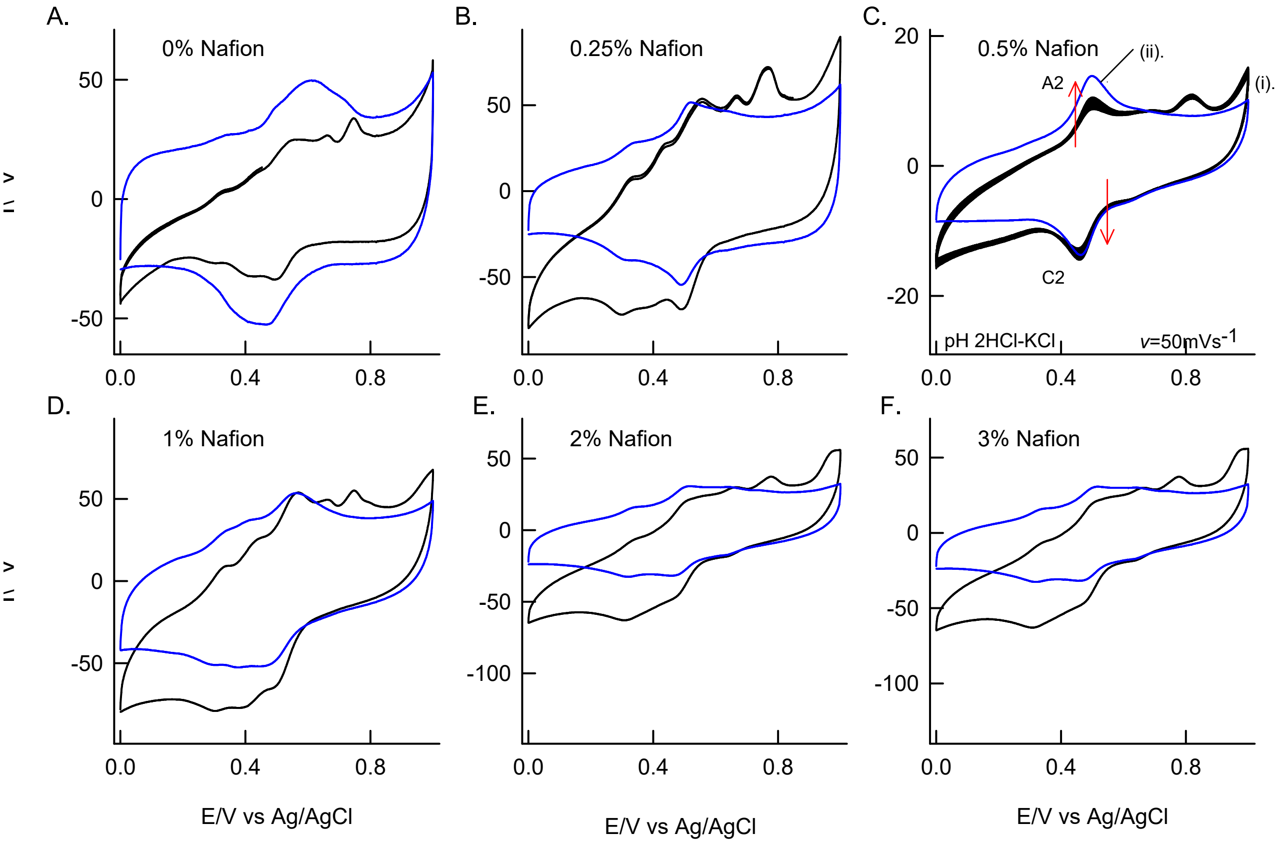

Supplementary Fig. S2. (A-E) CV responses of GCE/MWCNT in 50mM Ph-OCH_3-Redox+9mM H_2O_2 with various concentrations of Nafion (0% to 3%) (i) and its medium transferred responses ((ii); GCE/MWCNT@Ph-OCH_3-Redox) in pH 2 KCl-HCl buffer at *v*= 50 mV s^{-1}.

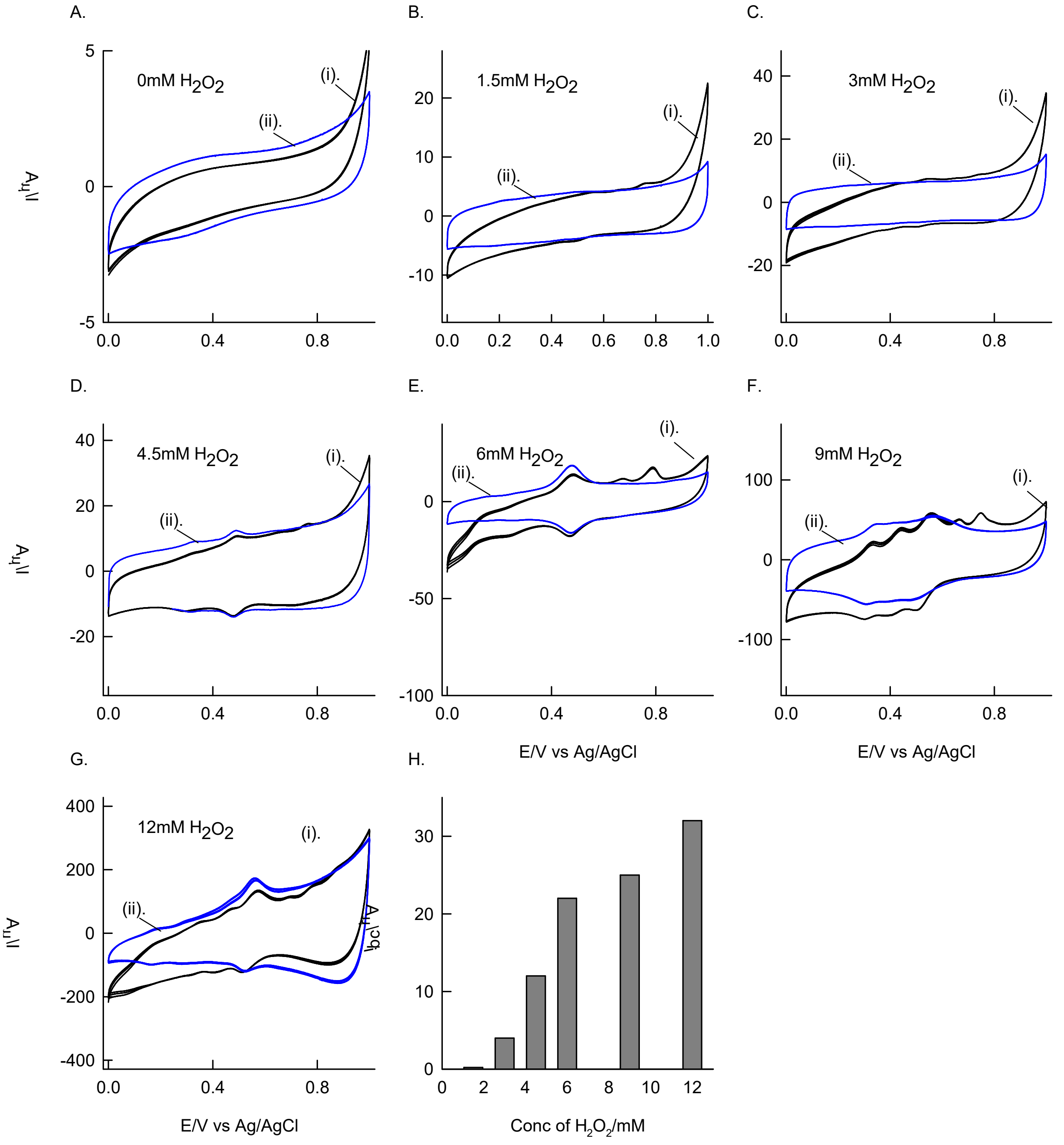

Supplementary Fig. S3. (A-G) CV responses of GCE/Nf-MWCNT exposed 50mM $PhOCH_3$-Redox+ x mM H_2O_2 (curve (i)) and its medium transferred responses (curve (ii); GCE/Nf-MWCNT@ $PhOCH_3$-Redox) in pH 2 KCl-HCl buffer at v= 50 mV s^{-1} and (H) its comparative plot of reduction peak current vs different concentration of H_2O_2..

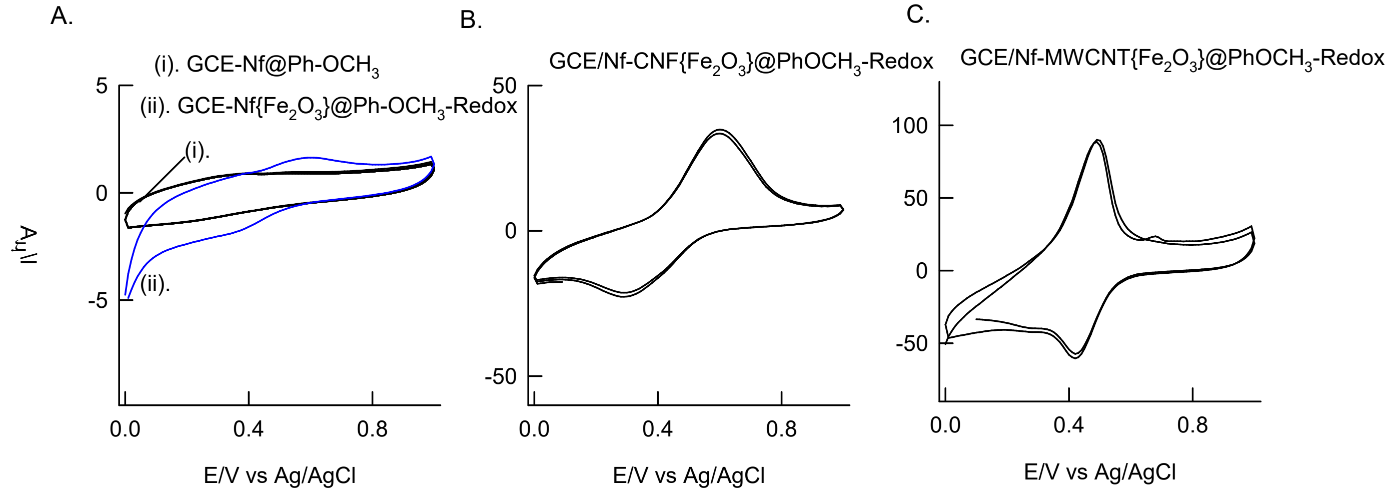

Supplementary Fig. S4. CV responses of (A) GCE-Nf@PhOCH$_3$-Redox (curve (i)) and GCE-Nf/Fe$_2$O$_3${Fe$_2$O$_3$}@PhOCH$_3$-Redox (curve (ii)), (B) GCE/Nf-CNT{Fe$_2$O$_3$}@PhOCH$_3$-Redox and (C) GCE/N-MWCNT{Fe$_2$O$_3$}@PhOCH$_3$-Redox in KCl-HCl buffer at v= 50 mV s^{-1}. {Fe$_2$O$_3$}=Deliberately added iron oxide to the carbon matrix.

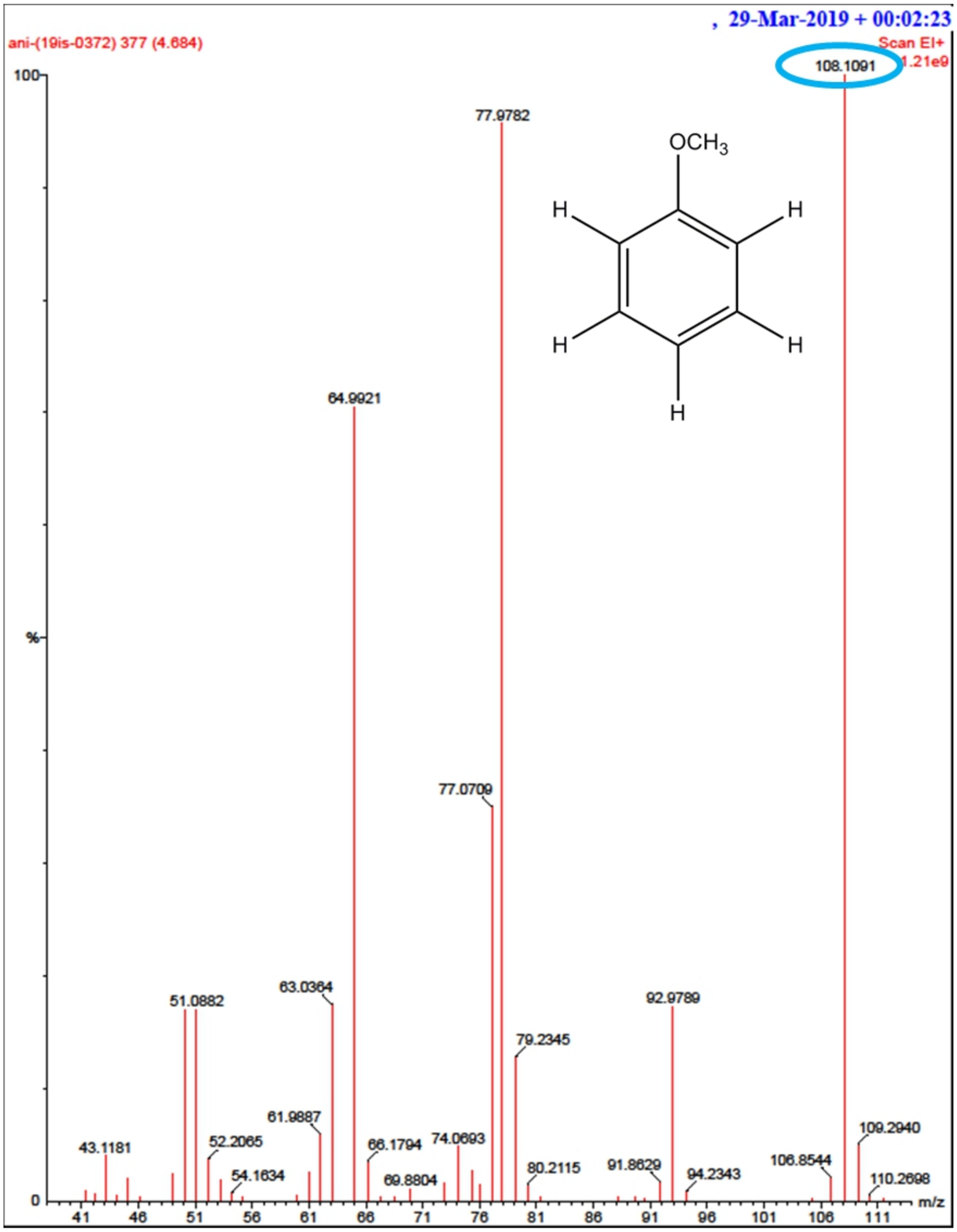

Supplementary Fig. S5A. Control GC-MS response of 108.14, $PhOCH_3$ (calculated molecular weight 108.14).

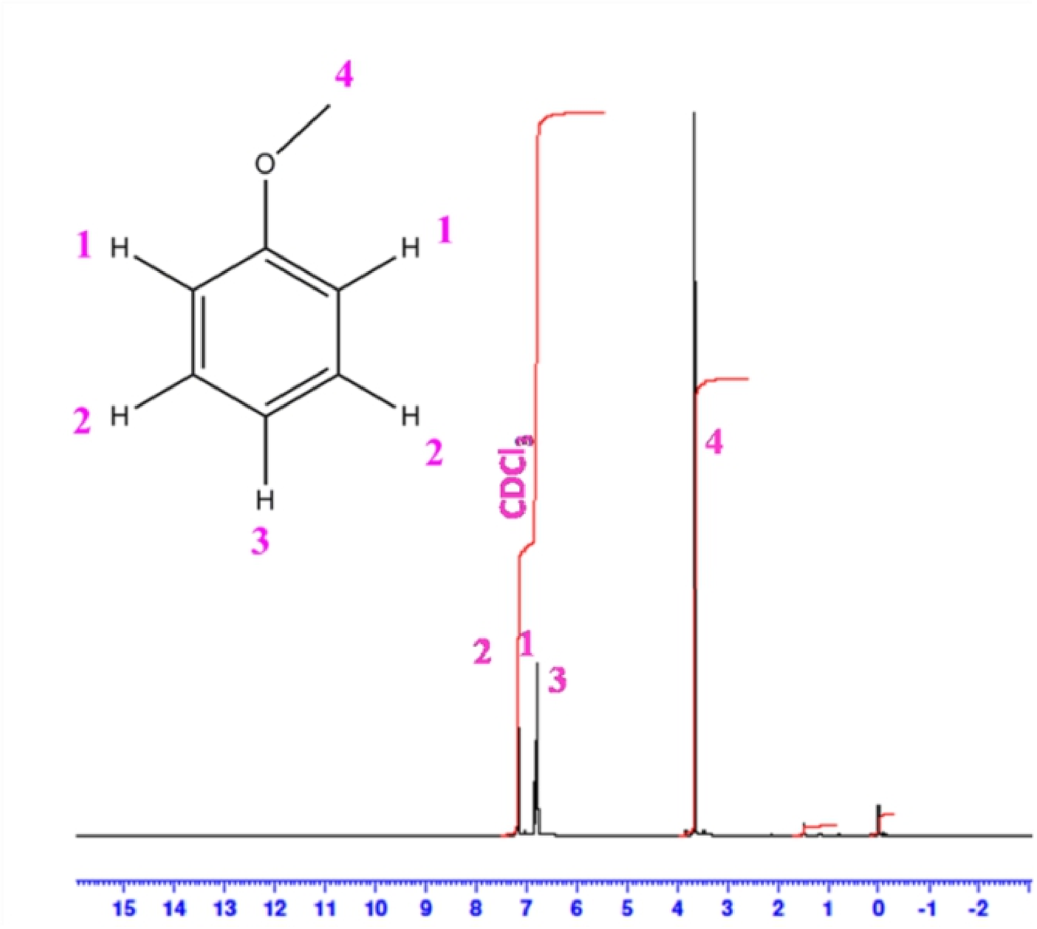

Supplementary Fig. S5B. Control NMR spectra of the $PhOCH_3$ (B, control) using $CDCl_3$ as a solvent.

A.

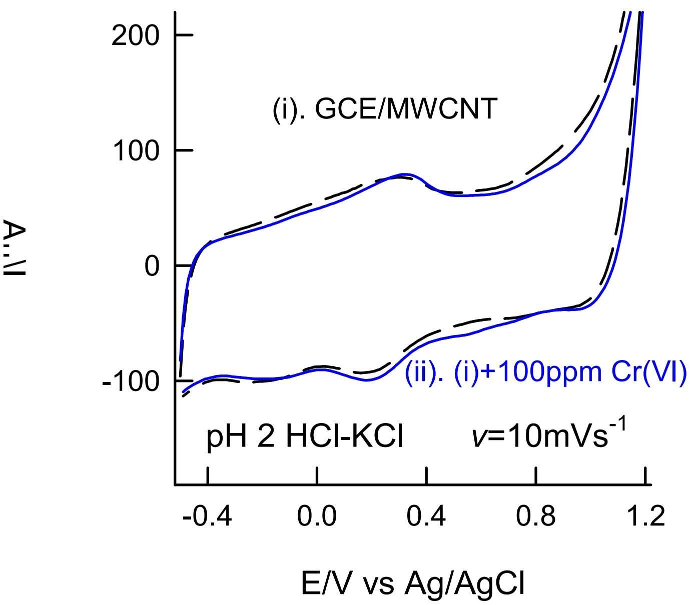

Supplementary Fig. S6. Control Experiments for CV response with various systems of GCE/MWCNT (A) without (curve (i)) and with 100ppm Cr(Vl) (curve (ii)) dissolved in pH 2 KCl-HCl buffer at *v*= 10 mV s^{-1}.

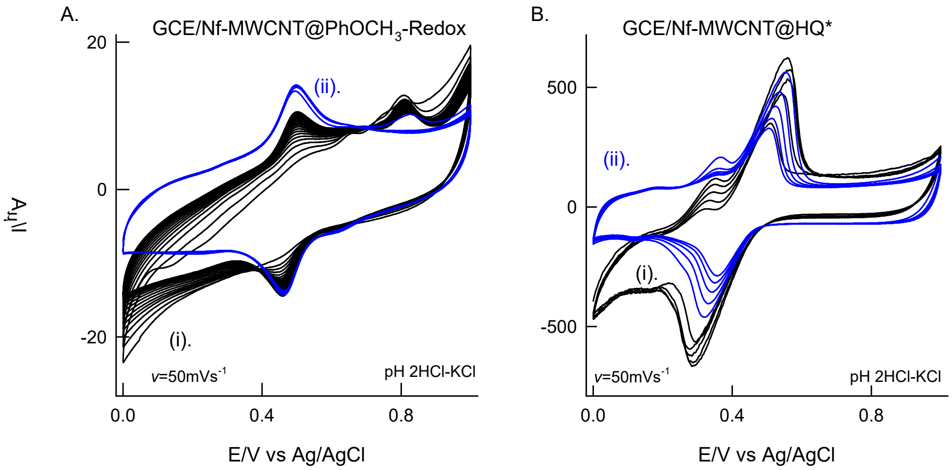

Supplementary Fig. S7. Comparative control experiments for ten continuous CV responses of GCE/Nf-MWCNT with 50 mM $PhOCH_3$-Redox (A), 10mM Hydroquinone (B) in pH buffer (curve (i)) and its medium transferred responses (curve (ii)) in pH 2 HCl-KCl at v= 50 mV s^{-1}.
